# Comparative Patho-Genomics of *Salmonella enterica* Serovar Enteritidis Reveal Potential Host-Specific Virulence Factors

**DOI:** 10.3390/pathogens14020128

**Published:** 2025-02-01

**Authors:** Matthew R. Moreau, Lekshmi K. Edison, Yury V. Ivanov, Dona Saumya S. Wijetunge, Eranda Mangala K. Kurundu Hewage, Jessica E. Linder, Subhashinie Kariyawasam

**Affiliations:** 1 Department of Biology, Providence College, Providence, RI 02918, USA; mattm32486@gmail.com; 2 Department of Veterinary and Biomedical Sciences, Pennsylvania State University, University Park, PA 16802, USA; yivanov.psu@gmail.com (Y.V.I.); dona.wijetunge@outlook.com (D.S.S.W.); eramkvet@gmail.com (E.M.K.K.H.); jessica.e.linder@gmail.com (J.E.L.); 3 Department of Comparative Diagnostics and Population Medicine, University of Florida, Gainesville, FL 32608, USA; edison.le@ufl.edu; 4 Houston Health Department, Houston, TX 77054, USA; 5 Immatics Biotechnologies, Houston, TX 77477, USA; 6 College of Veterinary Medicine, Purdue University, West Lafayette, IN 47907, USA

**Keywords:** *Salmonella* Enteritidis, virulence factors, host-specificity, comparative genomics, pathogenicity islands (SPI-1 and SPI-2)

## Abstract

*Salmonella enterica* serovar Enteritidis (*S.* Enteritidis) is one of the most common causes of bacterial foodborne infections worldwide. It has an extensive host range, including birds and humans, making it one of the most adaptable *Salmonella* serovars. This study aims to define the virulence gene profile of *S.* Enteritidis and identify genes critical to its host specificity. Currently, there is limited understanding of the molecular mechanisms that allow *S.* Enteritidis to continue as an important foodborne pathogen. To better understand the genes that may play a role in the host-specific virulence and/or fitness of *S.* Enteritidis, we first compiled a virulence gene profile-based genome analysis of sequenced *S.* Enteritidis strains isolated from shell eggs in our laboratory. This analysis was subsequently used to compare the representative genomes of *Salmonella* serovars with varying host ranges and *S.* Enteritidis genomes. The study involved a comprehensive and direct examination of the conservation of virulence and/or fitness factors, especially in a host-specific manner—an area that has not been previously explored. Key findings include the identification of 10 virulence-associated clusters of orthologous genes (COGs) specific to poultry-colonizing serovars and 12 virulence-associated COGs unique to human-colonizing serovars. Virulence/fitness-associated gene analysis identified more than 600 genes. The genome sequences of the two *S.* Enteritidis isolates were compared to those of the other serovars. Genome analysis revealed a core of 2817 COGs that were common to all the *Salmonella* serovars examined. Comparative genome analysis revealed that 10 virulence-associated COGs were specific to poultry-colonizing serovars, whereas 12 virulence-associated COGs were present in all human-colonizing serovars. Phylogenetic analyses further highlight the evolution of host specificity in *S*. Enteritidis. This study offers the first comprehensive analysis of genes that may be unique to and possibly essential for the colonization and/or pathogenesis of *S.* Enteritidis in various and specific hosts.

## 1. Introduction

*Salmonella enterica* comprises a group of Gram-negative, facultatively anaerobic, invasive, intracellular pathogens consisting of six subspecies and over 2600 serovars [1,2,3]. The subspecies of *Salmonella enterica* include *enterica*, *salamae*, *arizonae*, *diarizonae*, *houtenae*, and *indica* [4]. Among these, the subspecies *enterica* (*S. enterica*) is very well-adapted and capable of causing both gastrointestinal tract (GIT) (non-typhoidal) and systemic (typhoid, paratyphoid or typhoid-like) infections in a wide range of avian and mammalian species [5]. The *S. enterica* serovars that exhibit a multi-host range (i.e., broad-host range and host-adapted) include *Salmonella* Typhimurium, *Salmonella* Enteritidis, *Salmonella* Choleraesuis, *Salmonella* Heidelberg, *Salmonella* Paratyphoid-B, and many others [6,7]. Among these multi-host serovars, *S.* Typhimurium and *S.* Enteritidis are the two most common sources of foodborne salmonellosis worldwide [8,9,10]. In fact, recent epidemiological studies by the World Health Organization (WHO) and the Centers for Disease Control and Prevention (CDC) suggest that *S.* Enteritidis is a predominant source of bacterial foodborne illnesses in humans [11,12].

Despite various countermeasures and an overall decline in the prevalence of *S.* Enteritidis in chicken farms, this pathogen continues to pose a significant threat to human health [13,14]. *S.* Enteritidis infections in humans typically present with vomiting, diarrhea, abdominal cramping, fever, and other symptoms of gastroenteritis, which usually appear 12–72 hours after infection. Immunocompromised individuals, as well as those with weakened immune systems, such as the very young and elderly, are at the highest risk of developing severe, invasive infections and other complications [15]. In all cases, if dehydration is left untreated or bacteria become systemic, the illness can be fatal; however, this remains a relatively rare occurrence [16,17].

In contrast, commercial layer chickens are often asymptomatic when colonized by *S.* Enteritidis and can carry the bacterium undetected [8]. This allows *S.* Enteritidis to persist in hens and contaminate shell eggs, thus making contaminated eggs a common source of *S.* Enteritidis infections in humans. It is estimated that approximately one in every 20,000 eggs may be contaminated with *S.* Enteritidis. Given that over 65 billion eggs are produced annually in the United States alone, this translates to approximately 3.25 million contaminated eggs. Additionally, recent data indicate an increasing prevalence of *S.* Enteritidis in broiler chicken populations as well [18,19].

*S.* Enteritidis shares a high degree of genetic relatedness with many other *S. enterica* serovars [5,20,21]. However, few studies have compared multiple *S. enterica* serovars to *S.* Enteritidis, and even these studies have not defined a potential virulence/fitness gene profile or identified genes based on their ability to colonize specific hosts or contribute to the central pathogenicity of *S. enterica* [22,23,24,25]. Prior to the next-generation sequencing era, pulsed-field gel electrophoresis (PFGE) was considered the gold standard method for typing *Salmonella* to study its genetic relatedness [26]. In this study, we isolated two *S*. Enteritidis strains, designated SEE1 and SEE2, from chicken shell eggs. Both strains belong to the PFGE type JEGX01.0004, which is the most common PFGE fingerprint pattern associated with human *S.* Enteritidis foodborne illness [27]. To our knowledge, whole genome sequencing combined with the prediction of a virulence/fitness gene profile has not been conducted for any isolates of *S.* Enteritidis belonging to the JEGX01.0004 PFGE type or for any *S.* Enteritidis isolated from shell eggs. Developing a predicted virulence/fitness gene profile for *S.* Enteritidis of this PFGE type is essential for understanding the genetic basis behind the high prevalence of human *S.* Enteritidis infections associated with this fingerprint pattern. Additionally, this study provides valuable insights into the genes and mechanisms underlying the overall pathobiology of *S.* Enteritidis. The present study aims to define a predicted virulence/fitness gene profile, identify genes involved in pathogenesis, and explore the genes critical to the host specificity of *S.* Enteritidis.

## 2. Materials and Methods

### 2.1. Bacterial Strains

*S.* Enteritidis strains (SEE1 and SEE2) isolated from chicken shell eggs at the Animal Diagnostic Laboratory, Pennsylvania State University (University Park, PA, USA), were sequenced and used in the study. The NCBI GenBank accession numbers for these two isolates, along with the other strains used in the comparative genomics analysis, are provided in Table 1.

### 2.2. Genome Annotation

The completed genomes were submitted to Rapid Annotation using the Subsystem Technology (RAST) version 2.0 server [28]. Genome outputs were analyzed using the Artemis genome browser [29] and manually annotated to assign gene names and locus numbers. All open reading frames (ORFs) were further analyzed using BLASTp and BLASTn available at the National Center for Biotechnology Information (NCBI) website (https://blast.ncbi.nlm.nih.gov) (accessed on 17 October 2024) to verify the correct sizes of ORFs and to identify ORFs not previously recognized by RAST. Additionally, the genomes were submitted to the NCBI annotation pipeline for acceptance into GenBank under the accession numbers listed in Table 1.

### 2.3. Phylogenetic Analysis of S. Enteritidis Isolates and Other S. enterica Serovars

Twelve *S.* Enteritidis genomes, along with eight other *S. enterica* serovars (Table 1), were fragmented into 54 bp-long DNA sequences and mapped into the SEE1 genome using ssaha v2.5.4 [30]. A comparative genome analysis matrix was generated, where each column represented the genome of a single isolate, and each row represented a single gene. Only single nucleotide polymorphism (SNP) sites that were variable across all genomes were considered for constructing a phylogenetic tree. The resulting SNP alignment file was used as input for phylogenetic tree construction with RAxML v7.0.4, using a General Time Reversible (GTR) model with a gamma correction among site rate variation and ten random starting trees [31]. Additionally, a phylogenetic analysis was conducted using the Blast Ring Image Generator (BRIG) V.0.95 software, which aligns sequences and compares base similarity across the entire genome [32].

### 2.4. Comparative Genome Analysis

Comparative genome analysis tables were generated using data from RAST and manual annotations (Supplementary Tables S1–S14). The sortable table of orthologous gene clusters was first filtered using only entries with a “1” across all serovars, resulting in a table of orthologs shared by all serovars. For genes relevant to poultry infection, the “1” rule was applied to SEE1, SEE2, *S.* Gallinarum, *S.* Pullorum, *S.* Typhimurium, *S.* Paratyphi-B, and *S.* Enteritidis, while the “0” rule was applied to *S.* Paratyphi-A and *S.* Typhi. For human infection, the same matrix rules were applied, except that *S.* Paratyphi-A and *S.* Typhi were assigned “1”, while *S.* Gallinarum and *S.* Pullorum were assigned “0”. The tables were then classified into three categories: (i) core gene families, (iia) shared gene families of poultry infecting *S. enterica* serovars, and (iib) shared families of human-infecting *S. enterica* serovars. Genes from these categories were compared side-by-side to ensure that there was no overlap. The resulting tables were further analyzed for genes with potential virulence-associated roles. The locus tags in the sortable comparative genome analysis tables (Supplementary Tables S1–S14) were based on the RAST and manual annotation. The corresponding COG numbers link each locus tag in SEE1/SEE2 to its locus position in the genomes of the other serovars. While the locus tags differed from those in the NCBI files, the gene coordinates remained consistent.

### 2.5. Predicted Virulence/Fitness Gene Profiling

The genes of SEE1 and SEE2 were manually analyzed following annotation using BLASTp and BLASTn, and Protein Family Alignment Model (PFAM) analysis. Genes were included in the virulence gene profile if they met one or more of the following criteria: (1) directly involved in virulence, (2) involved in the biogenesis of virulence-related particles, and/or (3) involved in accessing or acquiring molecules through pathogenic processes. Additionally, antigens located on the cell surface and hypothetical/putative genes with predicted products exported from the cell or functioning as effectors were included. The genes listed in the virulence gene profile tables originated from the original RAST/manual annotation (Supplementary Tables S3–S11 and S14). These genes maintain the same coordinates in the NCBI files, although the names/locus tags may differ between the annotations.

### 2.6. Orthologous Gene Clusters and Comparative Genome Matrices

Extracted coding sequences from *Salmonella* genomes, listed in Table 1, were compared using reciprocal all-against-all BLASTp [33]. Gene families or COGs were identified using OrthoMCL with an E-value cutoff of 1 × 10^−5^, at least 75% length coverage, and a minimum of 50% protein identity. The identified COGs were further clustered using the Markov cluster algorithm [34]. The unclustered gene families without BLAST hits were considered strain-specific gene families (unique genes). The results from OrthoMCL were used to generate comparative genomic matrices, which were categorized into redundant and non-redundant datasets. The non-redundant dataset was utilized for further analysis. The comparative genome non-redundant matrix was constructed with each column representing a genome and each row representing a gene family. The matrix entry (i,j) is “1” if gene family “i” is present in genome “j”, and “0” if it is absent. This sortable table enables the querying of the following: (i) “core gene families” present in all genomes, (ii) “shared gene families” found in the selected genomes of interest, and (iii) “unique genes” specific to individual strains.

## 3. Results

### 3.1. Overview of SEE1 and SEE2 Genomes 

**Figure 1:**
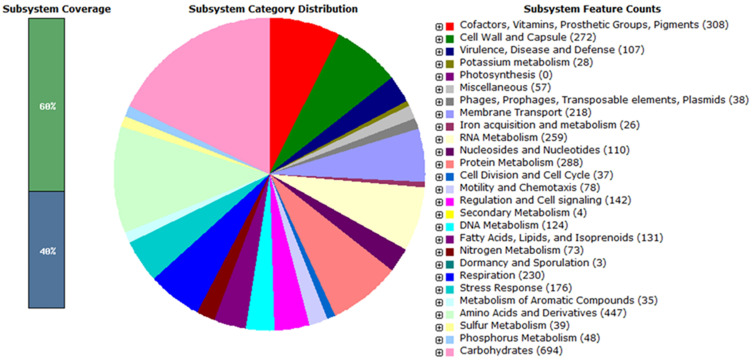
RAST genome profiling. The genomes of SEE1 and SEE2 were initially analyzed using RAST profiling. The RAST genome profile provides an approximate breakdown of the various gene subsystems present within the genome and the percentage of genes associated with each biochemical pathway. As shown in the left panel, 60% of the genes in the genome were classified as part of a specific subsystem, whereas 40% remained uncharacterized within any known subsystem (40%).

The genomes of both SEE1 and SEE2 were submitted to RAST for annotation and subsystem analysis. As shown in Figure 1, 60% of the genes in the genomes were associated with specific biochemical pathways and/or subsystems. The two largest subsystems identified were carbohydrate utilization and amino acid metabolism and synthesis. Both SEE1 and SEE2 genomes also contained genes associated with pathogenesis, with the most abundant subsystems being virulence, disease, and defense. As shown in Supplementary Table S1, nine genes in SEE1 and ten genes in SEE2 exhibited frameshift mutations. All the genes with frameshifts were identical between isolates, except for *ybiF*, which showed a frameshift in SEE2 but not in SEE1. Of these frameshifted genes, only *slrP* was relevant to the pathogenicity of *S*. Enteritidis (Supplementary Table S1).

### 3.2. Predicted Virulence/Fitness Gene Profiles of SEE1 and SEE2

**Figure 2:**
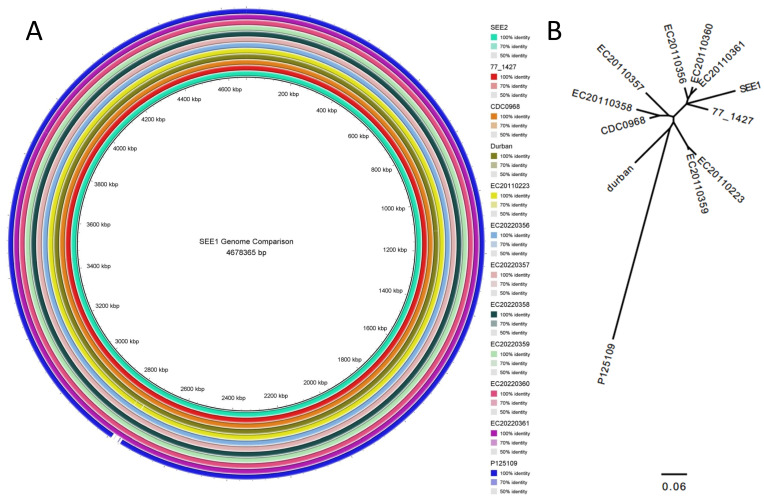
The phylogenetic relationship of SEE1 and SEE2 with human SE isolates. Two types of phylogenetic analyses were performed to compare the genomes of SEE1/SEE2 and 11 human *S.* Enteritidis isolates from GenBank. (**A**) The first analysis assessed whole-genome similarity based on sequence and gene presence/absence using the BRIG V.0.95 tool. The degree of similarity is represented by the shade of color (right panel) corresponding to each strain. The base color indicated 100% similarity, with lighter shades representing decreasing similarity. Less than 50% similarity, the general cutoff for other analyses, is marked in white to denote dissimilar loci. (**B**) The second analysis examined the whole-genome SNP content to evaluate genetic variation among the isolates.

To investigate the potential virulence gene profile of *S.* Enteritidis, we first compared the gene compositions of SEE1 and SEE2 with 11 selected human *S.* Enteritidis genome sequences from GenBank. As shown in Figure 2, SEE1 and SEE2 clustered very closely with all 11 genomes, both in genome content (Figure 2A) and whole-genome SNP comparisons (Figure 2B).

**Figure 3:**
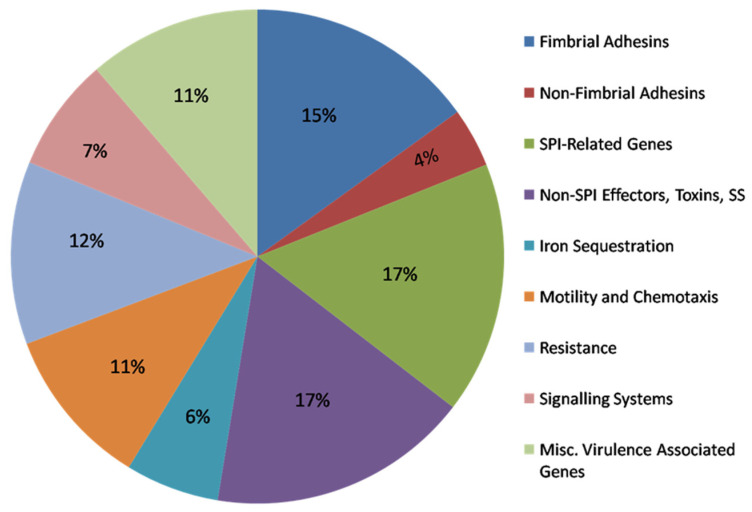
Composition breakdown of the virulence gene profiles of SEE1/SEE2. The percentage of genes in each predicted virulence/fitness gene class is represented by different colors, as shown above.

The predicted virulence/fitness gene profiles of SEE1 and SEE2 were developed based on the principle that these genes and their products may directly (e.g., effectors, toxins, and adhesins) or indirectly (e.g., nutrient acquisition systems and signaling) influence the virulence potential of *S.* Enteritidis. Using these criteria, both SEE1 and SEE2 were found to possess over 600 genes, accounting for approximately 13% of the total genome predicted to contribute to the virulence and fitness of *S.* Enteritidis. These categories include genes related to adherence, *Salmonella* Pathogenicity Islands (SPIs), non-SPI effectors, toxins, secretion systems, iron sequestration, motility and chemotaxis, antimicrobial resistance, signaling systems, and miscellaneous or putative virulence factors with unknown functions. These virulence/fitness genes and their annotations (functions) are listed in Supplementary Tables S3–S20, and the percentage of genes in each predicted virulence/fitness gene class is shown in Figure 3. It is important to note that the factors identified in these subsections (Figure 3 and Supplementary Tables S3–S20) are predictions and require further validation through in vitro and in vivo experiments to confirm their roles in virulence and/or fitness.

### 3.3. Genetic Relatedness of S. Enteritidis to Other S. enterica Serovars

**Figure 4:**
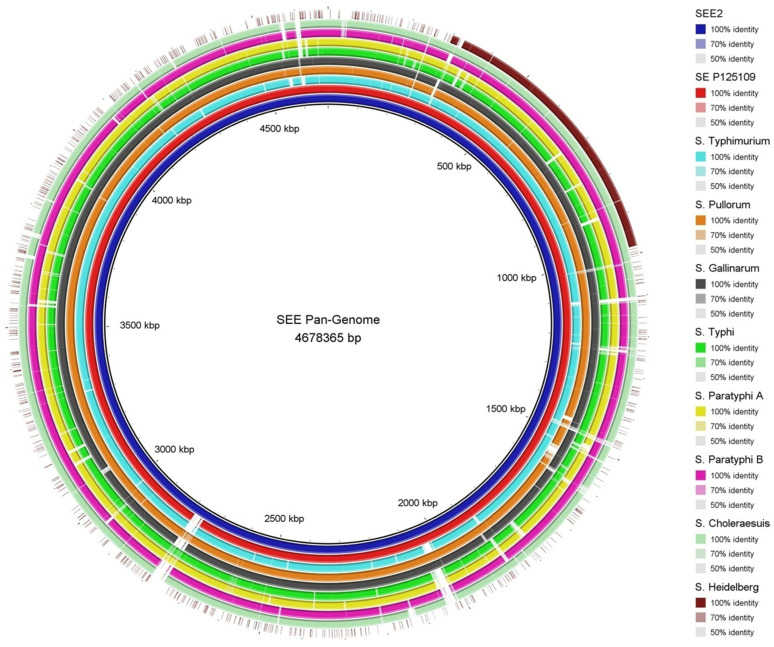
BRIG phylogenetic analysis of genome similarity of *Salmonella enterica* serovars. Whole genome similarities among the tested *S. enterica* serovars were analyzed using BRIG software V.0.95. The genomes were compared against the SEE1 genome backbone, and the results were visualized as inner-to-outer rings arranged by overall genome composition similarity. The degree of similarity is represented by the shade of color (right panel), where the base color corresponds to 100% similarity, and progressively lighter shades indicate decreasing similarity. Regions with less than 50% similarity, the cutoff for dissimilar loci in this analysis, are shown in white.

To better understand the evolution of the genome of *S*. Enteritidis, we aimed to determine how genetically related *S*. Enteritidis is compared to other serovars that can infect and/or colonize the same hosts. We conducted a whole-genome-based phylogenetic analysis of SEE1, SEE2, eight other serovars, and a canonical *S*. Enteritidis strain, P125109. These serovars included those restricted to poultry (*S*. Gallinarum and *S*. Pullorum), restricted to humans (*S*. Typhi and *S*. Paratyphi-A), and those capable of infecting or colonizing multiple hosts (*S*. Paratyphi-B, *S*. Typhimurium, *S*. Heidelberg, and *S*. Choleraesuis). The whole genome composition similarity analysis performed using BRIG revealed that SEE1 and SEE2 show the highest degree of similarity to the poultry-restricted *S*. Pullorum and *S*. Gallinarum, followed by *S*. Paratyphi-A, *S*. Paratyphi-B, *S*. Typhimurium, *S*. Choleraesuis, and finally with *S*. Heidelberg. As shown in Figure 4, there is a high conservation in most of the genomes of all the serovars compared to SEE1 and SEE2, except for *S*. Heidelberg, which exhibits a high degree of dissimilarity throughout its genome.

### 3.4. The Predicted Core and Host-Specific Virulence-Associated COGs

**Figure 5:**
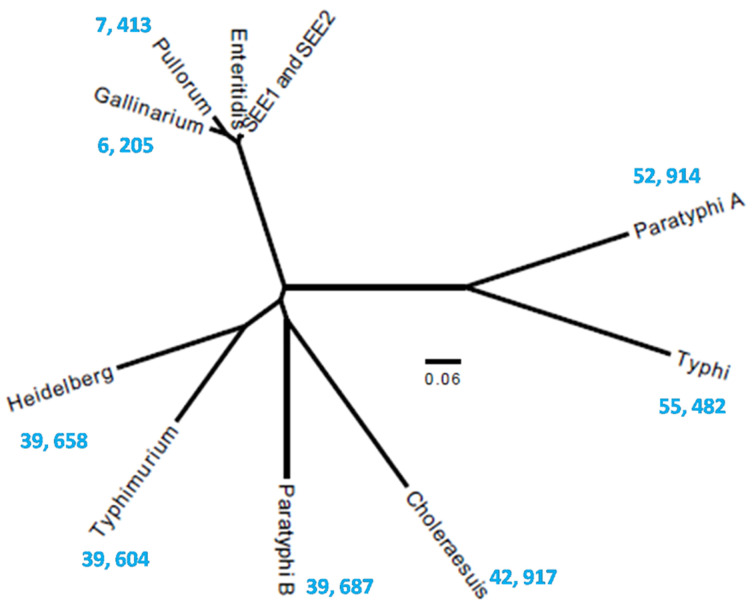
SNP-based phylogenetic analysis of *Salmonella enterica* serovars. The phylogenetic tree depicts the SNP-based genetic distances among the serovars calculated from SNPs in the conserved genes. The scale bar indicates the evolutionary distance between the serovars in base changes per site. To provide a clear perspective on genetic divergence, the total number of SNPs across the conserved genome was overlaid onto the tree, illustrating the variations in SNP counts between the serovars.

We then performed a genome-wide SNP analysis to compare the serovars (Figure 5). SEE1 and SEE2 clustered most closely with poultry-restricted serovars, followed by multi/broad-host range serovars, and were most distinctly related to human-restricted serovars. Among the conserved genes, fewer than 10,000 SNPs were observed between *S.* Enteritidis isolates and the poultry-restricted serovars. This number increased to between 39,000 and 43,000 SNPs when comparing *S.* Enteritidis with multi/broad-host range serovars and exceeded 50,000 SNPs when compared with human-restricted serovars.

**Figure 6:**
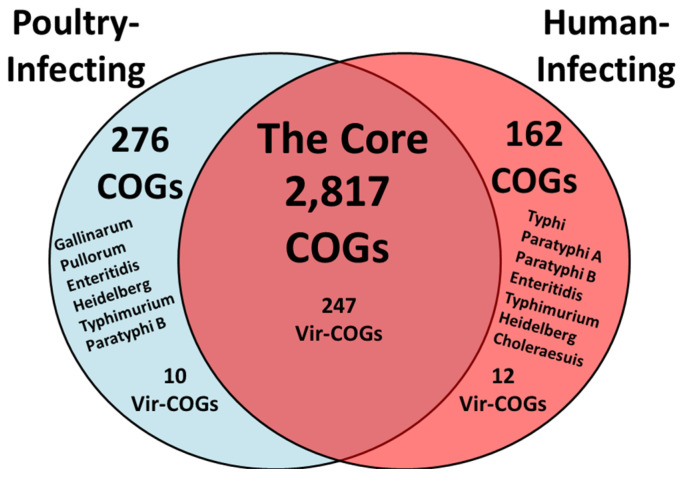
Venn diagram of the comparative genome analysis. The Venn diagram with clusters of COGs was categorized as follows: (i) COGs present in all serovars tested, representing the core genome, (ii) COGs specific to poultry infecting/colonizing serovars, excluding those restricted to humans (blue, left), and (iii) COGs specific to human infecting/colonizing serovars, excluding those restricted to poultry (red, right). The numbers within the diagram represent the genes predicted to be involved in the colonization/infection of specific hosts or conserved across *S. enterica* species.

Despite its broad host range, the predominant source of *S*. Enteritidis infections in humans is contaminated eggs and egg products. To investigate this, we searched for bioinformatics evidence of genes potentially involved in the colonization and/or infection of humans, chickens, and eggs, as well as genes that constitute the virulence repertoire of *S. enterica* as a species. As shown in Figure 6, the comparative-genome analysis revealed a total of 2817 COGs common to all tested serovars. To identify host-specific genes, we subtracted the genes found in human infecting/colonizing serovars to determine the COGs conserved in poultry infecting/colonizing serovars. Conversely, we applied the opposite approach to identify COGs enriched in serovars infecting/colonizing humans. The analysis identified 276 COGs that infect/colonize poultry and 162 COGs that were specific to those that infect/colonize humans. Here, we used one representative genome from each group because the average nucleotide identity (ANI) of the available genomes within each group exceeded 98%. The numbers of available genomes and their ANI values are listed in Table 2.

We cross-referenced the identified COGs with the previously developed virulence gene profile to create host-specific and species-specific predicted virulence/fitness gene profiles for *S*. Enteritidis. In total, 247 predicted virulence/fitness-related COGs were conserved among all serovars tested, including 70 associated with signaling and motility, 18 involved in lipopolysaccharide (LPS) biosynthesis, 59 encoding outer membrane proteins (OMPs) and adhesins, 20 related to transport, 26 linked to miscellaneous virulence functions, and 61 involved in the biosynthesis of SPI-related proteins (Supplementary Tables S15–S20). Among the 10 COGs specific to poultry infecting/colonizing serovars, four hypothetical genes exhibited structural similarities to virulence-associated genes. These included two outer membrane proteins (SEE1_0554 and SEE1_1013), a predicted SopD-like invasion effector (SEE1_0938), and a methyl-accepting chemotaxis protein (SEE1_1540), which functions as a receptor for galactose and ribose. Additionally, two sets of fimbrial operons containing genes *stiHBA* and *lpfDBA* were also identified (Table 3).

There were 12 predicted virulence/fitness-associated COGs specific to the human colonizing/infecting serovars that were identified in this study. Among these, four are associated with flagellar biosynthesis and assembly. The group included only one two-component system gene, *torS*, a hybrid histidine kinase. Interestingly, *iroD*, which encodes a salmochelin-specific esterase, was exclusively present in the human-infecting serovars and absent in *S.* Gallinarum and *S.* Pullorum. Another TonB-dependent iron transporter gene, *yncD*, was consistently found in these serovars. Additionally, two tetrathionate utilization genes, *ttrB* and *ttrC* were conserved within this group. The *sthA* gene, encoding a fimbrial chaperone protein, was the only fimbrial gene identified in these serovars. Furthermore, the *srfB* gene, part of the SsrAB regulon and located on the SPI-2 operon, was present in the human-infecting *S. enterica* serovars but absent in poultry-specific serovars (Table 4).

## 4. Discussion

*S.* Enteritidis remains a significant cause of human morbidity and mortality due to foodborne illness worldwide, including in the United States [35,36]. In humans, systemic illnesses are predominantly caused by infections with *S.* Typhi or *S.* Paratyphi, whereas in chickens and other avian species, *S.* Pullorum and *S.* Gallinarum are the most abundant serovars. However, the factors underlying these host-specific restrictions remain unclear [37,38]. The genomic analysis of SEE1 and SEE2 revealed over 600 genes that are potentially associated with virulence mechanisms, including motility, adherence, competition with microbiota, invasion, and intracellular growth and survival.

Among the 600 genes, several were associated with motility and signaling, iron acquisition, antimicrobial and other stress-related resistance, genes related to the SPIs, and genes with putative or miscellaneous functions (Figure 4 and Supplementary Tables S3–S14). These systems play a critical role in the pathogenesis and life cycle of *S.* Enteritidis and likely evolved as adaptations to maintain its broad host range and ability to cause disease. Defects in some of these systems could potentially restrict or entirely prevent *S.* Enteritidis from infecting specific host species. For example, poultry-restricted *S. Gallinarum* and *S. Pullorum* have deletions in their flagellar operons, rendering them pathogenic to poultry but unable to infect other species, at least in part due to the absence of functional flagella. This observation is consistent with previous studies suggesting that the loss of functional flagella contributes significantly to host specificity [25]. In SEE1 and SEE2, the *slrP*, which encodes an E-3 ubiquitin ligase ortholog, exhibits a major frameshift, likely rendering it a pseudogene (Supplementary Table S1) [39]. Despite this, both strains possess another E-3 ubiquitin ligase gene, *sseI*, which may function as the primary E-3 ligase, enabling these strains to retain their capacity to cause infection [40,41].

The evolutionarily conserved or lost functions of virulence genes have enabled *S.* Enteritidis to become a highly successful foodborne pathogen. For instance, SEE1 and SEE2 possess only two siderophore biosynthesis operons, which encode salmochelin and enterobactin, along with five ferric iron receptors (Supplementary Table S11). These systems are crucial for both the extracellular and intracellular phases of infection. Within eukaryotic cells, where iron is more abundant and competition for available iron is reduced, these two-siderophore systems may be sufficient for iron acquisition from the host. Conversely, multiple iron transporters may be vital during the extracellular phase, allowing *S.* Enteritidis to utilize siderophores secreted by *Salmonella* and other bacteria. Similarly, quorum sensing systems, conserved in extracellular enteric pathogens like *E. coli*, are also present in *S.* Enteritidis and other *S. enterica* serovars (Supplementary Tables S12 and S19). Recent studies have shown that quorum sensing plays a significant role in regulating SPI-1 and SPI-2, similar to the regulation of pathogenicity-associated islands (PAIs), such as the LEE operon in *E. coli*. These findings underscore the role of quorum sensing in the extracellular phase of infection and bacterial survival, consistent with existing studies on enteric pathogens [42,43,44,45].

The genomes of human-restricted serovars (*S.* Typhi and *S.* Paratyphoid), poultry-restricted serovars (*S.* Gallinarum and *S.* Pullorum), and serovars with multi- and broad-host ranges (*S.* Typhimurium, *S.* Heidelberg, *S.* Choleraesuis, and *S.* Enteritidis from various sources, including eggs) were compared to identify the genes critical for *S.* Enteritidis colonization and disease in their respective hosts. A genome-wide conserved gene SNP analysis was performed to further understand the phylogenetic relationship between *S.* Enteritidis and other serovars (Figure 5). These analyses revealed that *S.* Enteritidis shares more genes with serovars capable of infecting multiple hosts, while genome-wide gene conservation decreases as host specificity increases. Interestingly, the SNP-based clustering of conserved genes showed that *S.* Enteritidis clusters closer to poultry-restricted serovars, followed by multi-host serovars, and finally, human-restricted serovars. This clustering pattern aligns with previous phylogenetic studies that suggest evolutionary adaptations in *S*. Enteritidis for host specificity. [46].

In this study, two genomes of *S.* Enteritidis isolated from shell eggs were compared with 11 *S.* Enteritidis genomes and eight other *Salmonella* serovars capable of infecting or colonizing humans or poultry to identify potential virulence factors conserved across these serovars. The rationale behind this experimental design was that genes conserved between host-specific and multi-host range serovars may play essential roles in host-specific infection processes. The analysis identified 10 predicted virulence/fitness-associated genes specific to poultry-infecting serovars, which may contribute to the ability of *S.* Enteritidis to colonize poultry hosts (Table 3 and Figure 4). Among these, two sets of fimbrial genes, the *sti-*, and *lpf-*, are present in SEE1 and SEE2 and other poultry-infecting *S. enterica* serovars. The *lpf-* operon encodes long polar fimbriae, which may facilitate the attachment of *S.* Enteritidis to specific niches in poultry and/or shell eggs. Previous studies demonstrated that *lpf*-deficient *S.* Typhimurium was completely unable to form biofilms or attach to chicken epithelial cells, with only an intermediate loss of these traits when interacting with human HEp-2 cells [47]. This underscores how predicted virulence gene comparisons can provide biologically relevant insights into bacterial pathogenic processes.

A similar trend was observed in *S. enterica* isolates infecting humans, with 12 virulence-associated genes exclusively found in human-infecting isolates (Table 4). *Salmonella enterica* serovars produce a modified enterochelin, salmochelin, which is resistant to lipocalin-2 [48,49]. Notably, the esterase component of the salmochelin operon, *iroD*, was detected only in human-infecting isolates, suggesting that salmochelin has specifically evolved to facilitate iron acquisition in humans and mammals but not in poultry. Additionally, a predicted *TonB*-dependent iron receptor gene was identified in human-infecting serovars, although its exact function remains unknown. Its exclusive presence in human-infecting serovars suggests it may serve as an important redundant pathway for iron acquisition in the mammalian host, further highlighting the role of iron acquisition strategies in the adaptation of *S. enterica* to different hosts [48].

Three flagellar biosynthesis and assembly genes are shared between *S.* Enteritidis and other human-infecting serovars. One gene is located on the *flg-* operon and two on the *flh-* operon. In contrast, poultry-restricted *S. enterica* isolates, such as *S.* Pullorum and *S.* Gallinarum, are known to have frameshifts in the flagellar genes, rendering them non-motile. This loss of motility is believed to contribute to their host restriction to poultry. Additionally, the complete operon for the tetrathionate reductase complex is present in all human-infecting serovars, while two of its genes (*ttrB* and *ttrC*) are absent in poultry-restricted serovars. This suggests that tetrathionate utilization is more critical for the survival of *S.* Enteritidis in the mammalian gut than in poultry. This finding aligns with previous studies showing that the disruption of the microbiota in the mammalian gut releases tetrathionate into the GIT lumen, providing a nutrient advantage for *S. Enteritidis*. In contrast, infections with *S.* Enteritidis in poultry result in minimal changes to the cecal microbiota, suggesting a diminished importance of tetrathionate in the avian gut [50,51,52,53].

*Salmonella* SPI-1 and SPI-2 are essential for most *S. enterica* serovars to colonize various hosts and cause diseases in both poultry and humans. SPI-2 is regulated by multiple stimuli, some of which remain unidentified; however, PhoP/Q and SsrAB sensor kinase are two well-established regulators of this PAI [54,55,56]. Interestingly, the comparative genome analysis revealed that the COG associated with *srfB* (SsrAB-activated gene) is conserved only in human infecting isolates. This finding suggests that SPI-2 may be activated through an *SrfB*-independent mechanism in poultry and that SPI-2 is less critical (though still required) than SPI-1 for the successful colonization of poultry by *S.* Enteritidis [56,57,58]. This hypothesis is further supported by previous studies indicating that SPI-1 contributes more significantly to infection of poultry by *S. enterica* than SPI-2 [55].

Among the conserved COGs in *S. enterica*, three sets stand out as particularly interesting from an evolutionary perspective. These are the quorum sensing regulator QseBC (*qseBC*), the enterobactin iron sequestration system, and the tetrathionate reductase complex. The quorum sensing regulator QseBC is a well-characterized system that modulates virulence genes in other enteric pathogens, such as the LEE operon in enterohemorrhagic *E. coli*, through type 3 autoinducers like epinephrine and norepinephrine [59]. This system has also been shown to play an integral role in modulating *S. enterica* virulence in vivo [60]. The evolution of this system in *S.* Enteritidis appears to have been driven by adaptation to its various hosts because both mammals and birds produce the adrenaline hormones epinephrine and norepinephrine. These hormones are present in high concentrations in the GIT, which is the primary site of *Salmonella* colonization [59,60]. This evolutionary adaptation underscores the significance of quorum sensing in the virulence of *S. Enteritidis* and its interactions with the host.

*Salmonella enterica* can utilize tetrathionate as an alternative electron acceptor to support the breakdown of ethanolamine, enabling anaerobic respiration [51]. Our comparative pathogenomic analysis revealed that only the human infecting serovars retained all genes of the tetrathionate reduction complex. In mammals, *S.* Enteritidis can induce a shift in the gut microbiota, causing the release of tetrathionate through the inflammation of the host microenvironment while protecting itself from inflammatory processes by surviving intracellularly and producing superoxide dismutase [2,53]. This suggests that tetrathionate- and ethanolamine-driven anaerobic respiration is an adaptation for survival in mammals, as tetrathionate is less abundant in the poultry gut [52,53]. Interestingly, the conservation of ethanolamine utilization genes in poultry-specific serovars implies the presence of an unknown anaerobic electron acceptor in the poultry gut, distinct from tetrathionate. Another example of host-specific adaptation is that of enterobactin, which is conserved across all *S. enterica* serovars. Enterobactin can bind to and inactivate myeloperoxidase, a key immune defense mechanism in mammals, but this defense is present in much lower concentrations and with reduced efficacy in poultry heterophils [61]. While enterobactin has a greater affinity for iron than salmochelin, it is susceptible to sequestration and inactivation by lipocalin-2 in mammals, making salmochelin an essential adaptation for iron acquisition in hosts that produce lipocalin-2 [48]. Thus, the conservation of enterobactin across *S. enterica* serovars underscores its importance for survival in multiple hosts, while specialized modifications such as salmochelin represent evolutionary adaptations for specific host environments.

## 5. Conclusions

The divergence of host-specific and species-specific virulence factors in *S.* Enteritidis and other *S. enterica* serovars provides compelling evidence of the interplay between host microenvironments and pathogen genomic evolution. This study highlights the importance of these virulence factors in shaping host-pathogen interactions and their role in disease mechanisms. We believe that this new information will inspire further research into the functional roles of these genes at both the host and broader pathogenic levels. Such studies hold the potential to identify novel candidates for vaccines and antimicrobial therapies, which are critical for addressing the global health threat posed by *Salmonella* Enteritidis and other *Salmonella* serovars. These findings are especially relevant not only for combating established pathogens, but also for addressing emerging *Salmonella* strains that continue to pose challenges to animal and human health worldwide.

## Figures and Tables

**Table 1:** List of *S. enterica* strains used in this study.

Strain ID	Serovar	Accession Number	Date of Collection	Collection Location
SEE1	Enteritidis	CP011790.1 *	2010	Pennsylvania, USA
SEE2	Enteritidis	CP011791.1 *	2007	Pennsylvania, USA
P125109	Enteritidis	AM933172.1	1991	England, UK
EC20110360	Enteritidis	CP007258.1	2014	Alberta, Canada
EC20110359	Enteritidis	CP007259.1	2013	Alberta, Canada
EC20110358	Enteritidis	CP007260.1	2009	Alberta, Canada
EC20110357	Enteritidis	CP007261.1	2003	Alberta, Canada
EC20110356	Enteritidis	CP007262.1	2009	Alberta, Canada
EC20110361	Enteritidis	CP007263.1	2009	Alberta, Canada
EC20110223	Enteritidis	CP007266.1	2005	Alberta, Canada
Durban	Enteritidis	CP007507.1	2013	Durban, South Africa
CDC968	Enteritidis	CP007528.1	2010	Ohio, USA
77_1427	Enteritidis	CP007598.1	1977	Unknown, USA
SC-B67	Choleraesuis	AE017220.1	2002	Taiwan
C500	Choleraesuis	CP007639.1	2010	Yangzhou, China
ATCC 10708	Choleraesuis	CP012344.2	2018	Maryland, USA
A50	Choleraesuis	CM001062.1	1999	UK
287/91	Gallinarum	AM933173.1	2008	Brazil
RKS5078	Gallinarum	CP003047.1	2012	Brazil
SL476	Heidelberg	CP001120.1	2003	Minnesota, USA
B182	Heidelberg	CP003416.1	2012	France
CFSAN002069	Heidelberg	CP005390.2	2014	Washington, USA
41578	Heidelberg	CP004086.1	2011	Ohio, USA
ATCC 9150	Paratyphi A	CP000026.1	2014	Unknown
AKU_12601	Paratyphi A	FM200053.1	2002	Yemen
SPB7	Paratyphi B	CP000886.1	2007	Unknown
RKS4594	Paratyphi C	CP000857.1	1916	Norway
CDC1983-67	Pullorum	CP003786.1	2013	China
S06004	Pullorum	CP006575.1	2014	China
Ty2	Typhi	AE014613.1	1970	Russia
CT18	Typhi	AL513382.1	2001	Vietnam
Ty21a	Typhi	CP002099.1	1975	Egypt
P-stx-12	Typhi	CP003278.1	2012	India
LT2	Typhimurium	AE006468.1	1948	England, UK
ST4/74	Typhimurium	CP002487.1	1966	England, UK
UK-1	Typhimurium	CP002614.1	1991	England, UK
14028S	Typhimurium	CP001363.1	1960	England, UK
U288	Typhimurium	CP003836.1	2014	England, UK

* Note: The genomes of SEE1 and SEE2 were initially annotated using RAST and manual annotation. The accession numbers correspond to the NCBI GenBank database files, where the locus tags and coordinates are aligned with the same gene/locus tags provided in Supplementary Tables S1 and S2 for SEE1 and SEE2, respectively.

**Table 2:** Number of available genomes and ANI of *Salmonella* serovars. The ANI values indicated the genetic similarity among genomes within each group, which consistently exceeded 98%, validating the use of representative genomes for comparative genome analysis. This table highlights the high degree of conservation within each serovar group, providing a reliable basis for identifying host-specific and conserved COGs.

Salmonella Serovars	Number of Genomes Available	Average Nucleotide Identity (ANI) in %
*S.* Gallinarum	90	98.91
*S.* Pullorum	166	98.91
*S.* Enteritidis	40,234	98.9
*S.* Heidelberg	4750	99.14
*S.* Typhimurium	34,440	99.91
*S.* Paratyphi A	2726	98.55
*S.* Paratyphi B	2101	98.97
*S.* Typhi	9604	98.5
*S.* Choleraesuis	287	98.85

Note: The number of genomes was counted based on the available data in the NCBI database on 27 December 2024, and may vary with new submissions. ANI values above 95% generally indicate that the compared genomes belong to the same species, with higher percentages suggesting closer genetic relationships.

**Table 3:** Predicted poultry-specific *Salmonella enterica* virulence/fitness genes and their corresponding protein functions specific to *S. enterica* serovars are capable of infecting/colonizing poultry. These genes were identified through a comparative genome analysis that included all genes shared among serovars infecting/colonizing poultry (including poultry-specific and multi-host range serovars) while excluding the genes present in human-restricted serovars.

Gene	Description
*stiH*	fimbriae
*stiB*	fimbrial chaperone
*stiA*	fimbrial subunit
*SEE1_0554*	outer membrane protein
*SEE1_0938*	secreted protein SopD-like protein
*SEE1_1013*	outer membrane protein
*SEE1_1540*	chemotaxis protein–ribose–galactose sensor receptor
*lpfD*	long polar fimbrial operon protein
*lpfB*	long polar fimbrial chaperone
*lpfA*	long polar fimbria

**Table 4:** Predicted human-specific *Salmonella enterica* virulence/fitness genes and their corresponding protein functions specific to *S. enterica* serovars capable of infecting/colonizing humans. These genes were identified through a comparative genome analysis that included all genes shared among serovars infecting/colonizing humans (including human-specific and multi-host range serovars) while excluding genes present in poultry-restricted serovars.

Gene	Description
*yncD*	predicted TonB-dependent iron receptor
*flgK*	flagellar hook-associated protein FlgK
*ttrC*	tetrathionate reductase complex subunit C
*srfB*	ssrAB-activated gene
*flhA*	flagellar biosynthesis protein FlhA
*flhB*	flagellar biosynthesis protein FlhB
*pegC*	outer membrane protein
*iroD*	enterochelin esterase
*torS*	hybrid sensory histidine kinase TorS
*sthA*	fimbrial chaperone protein
*ttrB*	tetrathionate reductase complex subunit B
*sifB*	secreted effector protein

## Data Availability

The original contributions presented in this study are included in the article/Supplementary Materials. Further inquiries can be directed to the corresponding author.

## Supplementary Materials

The following supporting information can be downloaded at https://www.mdpi.com/article/10.3390/pathogens14020128/s1: Table S1: EE1/SEE2 Genes that Exhibit Frameshifts; Table S2: SEE1/SEE2 Genome Prophages; Table S3: SEE1/SEE2 Fimbrial Adhesins Genes; Table S4: SEE1/SEE2 Non-Fimbrial Adhesin Genes; Table S5: SEE1/SEE2 SPI-1 Related Genes; Table S6: SEE1/SEE2 SPI-II Related Genes; Table S7: SEE1/SEE2 SPI-III Related Genes; Table S8: SEE1/SEE2 SPI-IV Related Genes; Table S9: SEE1/SEE2 SPI-V Related Genes; Table S10: SEE1/SEE2 Non-SPI Related Toxins and Secretion System Genes; Table S11: SEE1/SEE2 Iron Sequestration Genes; Table S12: SEE1/SEE2 Signaling and Motility Genes; Table S13: SEE1/SEE2 Antimicrobial Resistance Genes; Table S14: SEE1/SEE2 Miscellaneous/Putative Virulence Genes; Table S15: Core Conserved Adherence and OMP COGs in *Salmonella enterica*; Table S16: Core Conserved SPI COGs in *Salmonella enterica*; Table S17: Core Conserved LPS Biosynthesis COGs in *Salmonella enterica*; Table S18: Core Conserved Transporter COGs in *Salmonella enterica*; Table S19: Core Conserved Signaling and Motility COGs in *Salmonella enterica*; Table S20: Core Conserved Miscellaneous/Survival COGs in *Salmonella enterica*.

## Abbreviations

The following abbreviations are used in this manuscript:

COGs	Clusters of orthologous genes;
GIT	Gastrointestinal tract;
WHO	World Health Organization;
CDC	Centers for Disease Control and Prevention;
PFGE	Pulsed-field gel electrophoresis;
RAST	Rapid Annotation using Subsystem Technology;
ORFs	Open reading frames;
SNP	Single nucleotide polymorphism;
BRIG	Blast Ring Image Generator;
NCBI	National Center for Biotechnology Information;
SPIs	*Salmonella* pathogenicity islands;
ANI	Average nucleotide identity;
LPS	Lipopolysaccharide;
OMPs	Outer membrane proteins;
PAIs	Pathogenicity-associated islands
