# Supplementary material for: Comparative Patho-Genomics of Salmonella enterica Serovar Enteritidis Reveal Potential Host-Specific Virulence Factors
Source: Pathogens. 2025 Feb 1;14(2):128. doi: 10.3390/pathogens14020128 (PMC11858713; doi:10.3390/pathogens14020128)
Supplement: Supplementary file 1 [file pathogens-14-00128-s001.zip › pathogens-3439838-supplementary.pdf]

Table S1. SEE1/SEE2 Genes that Exhibit Frameshifts

SEE1		SEE2	
**Gene**	**Description**	**Gene**	**Description**
slrP	invasion plasmid antigen / internalin, putative	*slrP*	invasion plasmid antigen / internalin, putative
agp	Glucose-1-phosphatase	*agp*	Glucose-1-phosphatase
SEE1_2169	putative cytoplasmic protein	*SEE2_2166*	putative cytoplasmic protein
SEE1_3845	ATP binding protein	*SEE2_3838*	ATP binding protein
yihO	Glucuronide transport protein YihP	*yihO*	Glucuronide transport protein YihP
idnT	L-idonate, D-gluconate, 5-keto-D-gluconate transporter	*idnT*	L-idonate, D-gluconate, 5-keto-D-gluconate transporter
mrr	FIG01047896: hypothetical protein	*mrr*	FIG01047896: hypothetical protein
ubiC	4-hydroxybenzoate polyprenyltransferase	*ubiC*	4-hydroxybenzoate polyprenyltransferase
SEE1_3889	Putative periplasmic protein	*SEE2_3882*	Putative periplasmic protein
		ybiF	

Table S2. SEE1/SEE2 Genome Prophages

Genes	Description	Genes	Description
SEE2_0279	Uncharacterized protein ImpA	*SEE2_0279*	Uncharacterized protein ImpA
SEE2_0280	Uncharacterized protein ImpH/VasB	*SEE2_0280*	Uncharacterized protein ImpH/VasB
SEE2_0281	Probable secreted protein	*SEE2_0281*	Probable secreted protein
SEE2_0282	Putative cytoplasmic protein	*SEE2_0282*	Putative cytoplasmic protein
SEE2_0283	VgrG protein	*SEE2_0283*	VgrG protein
SEE2_0284	Rhs-family protein	*SEE2_0284*	Rhs-family protein
SEE2_0285	FIG01046258: hypothetical protein	*SEE2_0285*	FIG01046258: hypothetical protein
SEE2_0286	FIG01046370: hypothetical protein	*SEE2_0286*	FIG01046370: hypothetical protein
SEE2_0287	FIG01046306: hypothetical protein	*SEE2_0287*	FIG01046306: hypothetical protein
SEE2_0288	rhs core protein with extension	*SEE2_0288*	rhs core protein with extension
SEE2_0289	hypothetical protein	*SEE2_0289*	hypothetical protein
SEE2_0290	FIG01046337: hypothetical protein	*SEE2_0290*	FIG01046337: hypothetical protein
SEE2_0291	putative cytoplasmic protein	*SEE2_0291*	putative cytoplasmic protein
SEE2_0292	FIG01046190: hypothetical protein	*SEE2_0292*	FIG01046190: hypothetical protein
SEE2_0293	Putative transposase	*SEE2_0293*	Putative transposase
SEE2_0294	No product	*SEE2_0294*	No product
SEE2_0295	Mobile element protein	*SEE2_0295*	Mobile element protein
safA	Fimbrial Lipoprotein	*safA*	Fimbrial Lipoprotein
safB	Periplasmic fimbrial chaperone protein	*safB*	Periplasmic fimbrial chaperone protein
safC	FIG034929: Fimbriae usher protein SafC	*safC*	FIG034929: Fimbriae usher protein SafC
safD	Putative fimbrial structural subunit	*safD*	Putative fimbrial structural subunit
ybeJ	Polysaccharide deacetylase	*ybeJ*	Polysaccharide deacetylase
SEE2_0301	Putative protein	*SEE2_0301*	Putative protein
SEE2_0302	TnpA	*SEE2_0302*	TnpA
SEE1_0975	Gifsy-2 prophage RecT	*SEE2_0973*	Gifsy-2 prophage RecT
SEE1_0976	FIG01046854: hypothetical protein	*SEE2_0974*	FIG01046854: hypothetical protein
SEE1_0977	FIG01046508: hypothetical protein	*SEE2_0975*	FIG01046508: hypothetical protein
SEE1_0978	Phage tail fiber protein	*SEE2_0976*	Phage tail fiber protein
SEE1_0979	Phage tail fibers	*SEE2_0977*	Phage tail fibers
sseI	Secreted effector protein	*sseI*	Secreted effector protein
SEE1_0981	Transposase	*SEE2_0979*	Transposase

SEE1_0982	Gifsy-2 prophage protein	*SEE2_0980*	Gifsy-2 prophage protein
SEE1_0983	FIG01047756: hypothetical protein	*SEE2_0981*	FIG01047756: hypothetical protein
SEE1_0984	FIG01046696: hypothetical protein	*SEE2_0982*	FIG01046696: hypothetical protein
SEE1_0985	hypothetical protein	*SEE2_0983*	hypothetical protein
SEE1_0986	Virulence protein msgA	*SEE2_0984*	Virulence protein msgA
SEE1_0987	hypothetical protein	*SEE2_0985*	hypothetical protein
SEE1_1216	Copper resistance protein D	*SEE2_1211*	Copper resistance protein D
SEE1_1217	Putative periplasmic or exported protein	*SEE2_1212*	Putative periplasmic or exported protein
SEE1_1218	Mobile element protein	*SEE2_1213*	Mobile element protein
SEE1_1219	excisionase	*SEE2_1214*	excisionase
SEE1_1220	Putative hydrolase	*SEE2_1215*	Putative hydrolase
SEE1_1221	hypothetical protein	*SEE2_1216*	hypothetical protein
SEE1_1222	FIG01047714: hypothetical protein	*SEE2_1217*	FIG01047714: hypothetical protein
SEE1_1223	Phage protein	*SEE2_1218*	Phage protein
SEE1_1224	Gifsy-2 prophage protein	*SEE2_1219*	Gifsy-2 prophage protein
SEE1_1225	antiterminator-like protein	*SEE2_1220*	antiterminator-like protein
SEE1_1226	hypothetical protein	*SEE2_1221*	hypothetical protein
SEE1_1227	FIG01048890: hypothetical protein	*SEE2_1222*	FIG01048890: hypothetical protein
SEE1_1228	hypothetical protein	*SEE2_1223*	hypothetical protein
SEE1_1229	GtgA	*SEE2_1224*	GtgA
SEE1_1230	Phage holin #Lambda-like group I holin	*SEE2_1225*	Phage holin #Lambda-like group I holin
SEE1_1231	Phage lysozyme	*SEE2_1226*	Phage lysozyme
SEE1_1232	Phage outer membrane lytic protein Rz; Endopeptidase	*SEE2_1227*	Phage outer membrane lytic protein Rz; Endopeptidase
SEE1_1233	FIG01049225: hypothetical protein	*SEE2_1228*	FIG01049225: hypothetical protein
SEE1_1234	Attachment invasion locus protein precursor	*SEE2_1229*	Attachment invasion locus protein precursor
sodC	Superoxide dismutase [Cu-Zn] precursor	*sodC*	Superoxide dismutase [Cu-Zn] precursor
SEE1_1236	Phage minor tail protein	*SEE2_1231*	Phage minor tail protein
SEE1_1237	Phage tail assembly protein	*SEE2_1232*	Phage tail assembly protein
SEE1_1238	Phage tail fiber protein	*SEE2_1233*	Phage tail fiber protein
SEE1_1239	Phage tail fiber protein	*SEE2_1234*	Phage tail fiber protein
SEE1_1240	Phage tail fibers	*SEE2_1235*	Phage tail fibers
SEE1_1241	invasion-associated secreted protein.	*SEE2_1236*	invasion-associated secreted protein.
SEE1_1242	DNA invertase from prophage CP-933H	*SEE2_1237*	DNA invertase from prophage CP-933H

SEE1_1243	FIG01045615: hypothetical protein	*SEE2_1238*	FIG01045615: hypothetical protein
SEE1_1244	Mobile element protein	*SEE2_1239*	Mobile element protein
SEE1_1245	FIG01047716: hypothetical protein	*SEE2_1240*	FIG01047716: hypothetical protein
SEE1_1246	Phage lysin, 1,4-beta-N-acetylmuramidase	*SEE2_1241*	Phage lysin, 1,4-beta-N-acetylmuramidase
SEE1_1247	Homology to phage-tail assembly proteins	*SEE2_1242*	Homology to phage-tail assembly proteins
SEE1_1248	lytic enzyme	*SEE2_1243*	lytic enzyme
SEE1_1249	FIG01046582: hypothetical protein	*SEE2_1244*	FIG01046582: hypothetical protein
SEE1_1250	FIG01045807: hypothetical protein	*SEE2_1245*	FIG01045807: hypothetical protein
SEE1_1251	FIG01047586: hypothetical protein	*SEE2_1246*	FIG01047586: hypothetical protein
SEE1_1252	FIG01046232: hypothetical protein	*SEE2_1247*	FIG01046232: hypothetical protein
SEE1_1253	Phage tail fiber protein	*SEE2_1248*	Phage tail fiber protein
mig-3	phage tail assembly-like protein	*mig-3*	phage tail assembly-like protein
SEE1_1255	Phage tail fiber protein	*SEE2_1250*	Phage tail fiber protein
SEE1_1256	Hypothetical protein	*SEE2_1251*	Hypothetical protein
SEE1_1257	Hypothetical protein	*SEE2_1252*	hypothetical protein
SEE1_1258	hypothetical protein	*SEE2_1253*	hypothetical protein
SEE1_1259	FIG01045658: hypothetical protein	*SEE2_1254*	FIG01045658: hypothetical protein
pagO	Inner membrane protein	*pagO*	Inner membrane protein
SEE1_1261	FIG01045706: hypothetical protein	*SEE2_1256*	FIG01045706: hypothetical protein
SEE1_1262	Hypothetical protein	*SEE2_1257*	hypothetical protein
SEE1_1263	Hypothetical protein	*SEE2_1258*	Hypothetical protein
SEE1_1264	Mobile element protein	*SEE2_1259*	Mobile element protein
SEE1_1265	Conserved secreted hypothetical protein	*SEE2_1260*	Conserved secreted hypothetical protein
SEE1_1266	hypothetical protein	*SEE2_1261*	hypothetical protein
SEE1_1267	Putative acetyltransferase	*SEE2_1262*	Putative acetyltransferase
SEE1_1268	FIG01045929: hypothetical protein	*SEE2_1263*	FIG01045929: hypothetical protein
SEE1_1269	Putative cytoplasmic protein	*SEE2_1264*	Putative cytoplasmic protein
SEE1_1270	FIG01045215: hypothetical protein	*SEE2_1265*	FIG01045215: hypothetical protein
SEE1_1271	G-nucleotide exchange factor SopE	*sopE2*	G-nucleotide exchange factor SopE
SEE1_1272	FIG01046404: hypothetical protein	*SEE2_1267*	FIG01046404: hypothetical protein

SEE1_1273	STY1986 from Accession AL513382: Salmonella typhi CT18	*SEE2_1268*	STY1986 from Accession AL513382: Salmonella typhi CT18
SEE1_1274	FIG01046004: hypothetical protein	*SEE2_1269*	FIG01046004: hypothetical protein
SEE1_1275	Ren protein	*prpA*	Ren protein
SEE1_1487	Phage integrase	*SEE2_1481*	Phage integrase
SEE1_1488	hypothetical protein	*SEE2_1482*	hypothetical protein
SEE1_1489	hypothetical protein	*SEE2_1483*	hypothetical protein
SEE1_1490	Kil protein	*SEE2_1484*	Kil protein
SEE1_1491	hypothetical protein	*SEE2_1485*	hypothetical protein
SEE1_1492	hypothetical protein	*SEE2_1486*	hypothetical protein
SEE1_1493	Mobile element protein	*SEE2_1487*	Mobile element protein
SEE1_1494	hypothetical protein	*SEE2_1488*	hypothetical protein
SEE1_1495	hypothetical protein	*SEE2_1489*	hypothetical protein
SEE1_1496	Putative protein	*SEE2_1490*	Putative protein
SEE1_1497	Unknown function	*SEE2_1491*	Unknown function
SEE1_1498	conserved hypothetical protein	*SEE2_1492*	conserved hypothetical protein
SEE1_1499	Phage protein	*SEE2_1493*	Phage protein
SEE1_1500	FIG00639062: hypothetical protein	*SEE2_1494*	FIG00639062: hypothetical protein
SEE1_1501	Phage antitermination protein Q	*SEE2_1495*	Phage antitermination protein Q
SEE1_1502	hypothetical protein	*SEE2_1496*	hypothetical protein
SEE1_1503	hypothetical protein	*SEE2_1497*	hypothetical protein
SEE1_1504	FIG00638630: hypothetical protein	*SEE2_1498*	FIG00638630: hypothetical protein
SEE1_1505	putative prophage membrane protein	*SEE2_1499*	putative prophage membrane protein
SEE1_1506	hypothetical protein	*SEE2_1500*	hypothetical protein
SEE1_1507	hypothetical protein	*SEE2_1501*	hypothetical protein
SEE1_1508	site-specific recombination	*SEE2_1502*	site-specific recombination
SEE1_1509	FIG01046477: hypothetical protein	*SEE2_1503*	FIG01046477: hypothetical protein
SEE1_2064	putative transposase	*SEE2_2058*	putative transposase
SEE1_2065	Mobile element protein	*SEE2_2059*	Mobile element protein
SEE1_2066	FIG01046422: hypothetical protein	*SEE2_2060*	FIG01046422: hypothetical protein
SEE1_2067	Hypothetical protein	*SEE2_2061*	Hypothetical protein
SEE1_2068	FIG01046824: hypothetical protein	*SEE2_2062*	FIG01046824: hypothetical protein
tRNA	tRNA-Ser-CGA	*tRNA*	tRNA-Ser-CGA
yeeI	FIG01220476: hypothetical protein	*yeeI*	FIG01220476: hypothetical protein
tRNA	tRNA-Asn-GTT	*tRNA*	tRNA-Asn-GTT

SEE1_2070	FIG01048042: hypothetical protein	*SEE1_2064*	FIG01048042: hypothetical protein
tRNA	tRNA-Asn-GTT	*tRNA*	tRNA-Asn-GTT
SEE1_2071	integrase	*SEE1_2065*	integrase
SEE1_2758	FIG01045174: hypothetical protein	*SEE2_2754*	FIG01045174: hypothetical protein
SEE1_2759	Large repetitive protein	*SEE2_2755*	Large repetitive protein
SEE1_2760	FIG01045638: hypothetical protein	*SEE2_2756*	FIG01045638: hypothetical protein
SEE1_2761	Putative type I secretion protein, ATP-binding protein	*SEE2_2757*	Putative type I secretion protein, ATP-binding protein
SEE1_2762	Putative HlyD family secretion protein	*SEE2_2758*	Putative HlyD family secretion protein
SEE1_2763	FIG01049483: hypothetical protein	*SEE2_2759*	FIG01049483: hypothetical protein
SEE1_2764	Gene D protein	*SEE2_2760*	Gene D protein
SEE1_2765	Phage tail protein	*SEE2_2761*	Phage tail protein
SEE1_2766	STY4603 from Accession AL513382: Salmonella typhi CT18	*SEE2_2762*	STY4603 from Accession AL513382: Salmonella typhi CT18
SEE1_2767	putative phage tail protein	*SEE2_2763*	putative phage tail protein
SEE1_2768	Tail protein	*SEE2_2764*	Tail protein
SEE1_2769	Phage major tail tube protein	*SEE2_2765*	Phage major tail tube protein
SEE1_2770	Phage major tail sheath protein	*SEE2_2766*	Phage major tail sheath protein
SEE1_2771	hypothetical protein	*SEE2_2767*	hypothetical protein
SEE1_2772	hypothetical protein	*SEE2_2768*	hypothetical protein
SEE1_2773	Phage tail fiber protein	*SEE2_2769*	Phage tail fibers
SEE1_2774	Tail fiber protein	*SEE2_2770*	Tail fiber protein
SEE1_2775	Phage tail fibers	*SEE2_2771*	Phage tail fibers
SEE1_2776	Baseplate assembly protein J	*SEE2_2772*	Baseplate assembly protein J
SEE1_2777	Phage baseplate assembly protein	*SEE2_2773*	Phage baseplate assembly protein
SEE1_2778	Baseplate assembly protein V	*SEE2_2774*	Baseplate assembly protein V
SEE1_2779	FIG01047449: hypothetical protein	*SEE2_2775*	FIG01047449: hypothetical protein
SEE1_2780	Phage tail completion protein	*SEE2_2776*	Phage tail completion protein
SEE1_2781	Phage tail protein	*SEE2_2777*	Phage tail protein
SEE1_2782	Phage spanin Rz	*SEE2_2778*	Phage spanin Rz
SEE1_2783	Phage lysin	*SEE2_2779*	Phage lysin
SEE1_2784	possible secretory protein	*SEE2_2780*	possible secretory protein
SEE1_2785	Phage tail X	*SEE2_2781*	Phage tail X
SEE1_2786	Phage head completion-stabilization protein	*SEE2_2782*	Phage head completion-stabilization protein
SEE1_2787	Phage terminase, endonuclease subunit	*SEE2_2783*	Phage terminase, endonuclease subunit

SEE1_2788	Phage major capsid protein	*SEE2_2784*	Phage major capsid protein
SEE1_2789	Phage capsid scaffolding protein	*SEE2_2785*	Phage capsid scaffolding protein
SEE1_2790	Phage terminase, ATPase subunit	*SEE2_2786*	Phage terminase, ATPase subunit
SEE1_2791	Phage capsid and scaffold	*SEE2_2787*	Phage capsid and scaffold
SEE1_2792	hypothetical protein	*SEE2_2788*	hypothetical protein
SEE1_2793	Hypothetical protein	*SEE2_2789*	Hypothetical protein
SEE1_2794	FIG00641226: hypothetical protein	*SEE2_2790*	FIG00641226: hypothetical protein
SEE1_2795	hypothetical protein	*SEE2_2791*	hypothetical protein
SEE1_2796	Phage replication protein	*SEE2_2792*	Phage replication protein
SEE1_2797	Phage replication protein	*SEE2_2793*	Phage replication protein
SEE1_2798	Methyl-directed repair DNA adenine methylase	*SEE2_2794*	Methyl-directed repair DNA adenine methylase
SEE1_2799	Phage protein	*SEE2_2795*	Phage protein
SEE1_2800	STY3665 from Accession AL513382: Salmonella typhi CT18	*SEE2_2796*	STY3665 from Accession AL513382: Salmonella typhi CT18
SEE1_2801	FIG01045453: hypothetical protein	*SEE2_2797*	FIG01045453: hypothetical protein
SEE1_2802	FIG00640946: hypothetical protein	*SEE2_2798*	FIG00640946: hypothetical protein
SEE1_2803	Regulatory protein CII	*SEE2_2799*	Regulatory protein CII
SEE1_2804	Phage regulatory protein	*SEE2_2800*	Phage regulatory protein
SEE1_2805	Phage repressor protein cI	*SEE2_2801*	Phage repressor protein cI
SEE1_2806	Phage integrase	*SEE2_2802*	Phage integrase
SEE1_2807	hypothetical protein	*SEE2_2803*	hypothetical protein
SEE1_2808	membrane protein	*SEE2_2804*	membrane protein
SEE1_2809	FIG01047617: hypothetical protein	*SEE2_2805*	FIG01047617: hypothetical protein
SEE1_2810	Hypothetical protein	*SEE2_2806*	Hypothetical protein
SEE1_2811	Mobile element protein	*SEE2_2807*	Mobile element protein
SEE1_2812	Mobile element protein	*SEE2_2808*	Mobile element protein
SEE1_2813	Mobile element protein	*SEE2_2809*	Mobile element protein
SEE1_2814	Hypothetical protein	*SEE2_2810*	Hypothetical protein
SEE1_2815	hypothetical protein SPUL_2764	*SEE2_2811*	hypothetical protein SPUL_2764

Table S3. SEE1/SEE2 Fimbrial Adhesins Genes

SEE1		SEE2	
**Gene**	**Description**	**Gene**	**Description**
bcfA	Type 1 fimbriae major subunit FimA	*bcfA*	Type 1 fimbriae major subunit FimA
bcfB	Chaperone FimC	*bcfB*	Chaperone FimC
papC	Type 1 fimbriae anchoring protein FimD	*papC*	Type 1 fimbriae anchoring protein FimD
bcfD	Fimbriae-like adhesin SfmH	*bcfD*	Fimbriae-like adhesin SfmH
bcfE	Type 1 fimbrae adaptor subunit FimF	*bcfE*	Type 1 fimbrae adaptor subunit FimF
bcfF	Type 1 fimbrae adaptor subunit FimF	*bcfF*	Type 1 fimbrae adaptor subunit FimF
bcfG	Hypothetical fimbrial chaperone ycbF precursor	*bcfG*	Hypothetical fimbrial chaperone ycbF precursor
hofC	Type IV fimbrial assembly protein PilC	*hofC*	Type IV fimbrial assembly protein PilC
hofB	Type IV fimbrial assembly, ATPase PilB	*hofB*	Type IV fimbrial assembly, ATPase PilB
ppd	Type IV pilin PilA	*ppd*	Type IV pilin PilA
stiH	Putative fimbriae	*stiH*	Putative fimbriae
stiC	FIG100795: Fimbriae usher protein StiC	*stiC*	FIG100795: Fimbriae usher protein StiC
stiB	Chaperone protein EcpD	*stiB*	Chaperone protein EcpD
stiA	Putative fimbrial subunit	*stiA*	Putative fimbrial subunit
stfA	Major fimbrial subunit StfA	*stfA*	Major fimbrial subunit StfA
stfC	Fimbriae usher protein StfC	*stfC*	Fimbriae usher protein StfC
stfD	Periplasmic fimbrial chaperone StfD	*stfD*	Periplasmic fimbrial chaperone StfD
stfE	Minor fimbrial subunit StfE	*stfE*	Minor fimbrial subunit StfE
stfF	Minor fimbrial subunit StfF	*stfF*	Minor fimbrial subunit StfF
stfG	Minor fimbrial subunit StfG	*stfG*	Minor fimbrial subunit StfG
safA	Putative fimbrial lipoprotein	*safA*	Putative fimbrial lipoprotein
safB	Periplasmic fimbrial chaperone protein	*safB*	Periplasmic fimbrial chaperone protein
safC	FIG034929: Fimbriae usher protein SafC	*safC*	FIG034929: Fimbriae usher protein SafC
safD	Putative fimbrial structural subunit	*safD*	Putative fimbrial structural subunit

crl	Curlin genes transcriptional activator	*crl*	Curlin genes transcriptional activator
stbE	Putative pilus chaperone, PapD family	*stbE*	Putative pilus chaperone, PapD family
stbD	Putative exported protein precursor	*stbD*	Putative exported protein precursor
stbC	outer membrane fimbrial usher protein	*stbC*	outer membrane fimbrial usher protein
stbB	Putative fimbrial chaperone	*stbB*	Putative fimbrial chaperone
stbA	Fimbrial protein precursor	*stbA*	Fimbrial protein precursor
fimA	Fimbriae-like adhesin SfmA	*fimA*	Fimbriae-like adhesin SfmA
fimI	Fimbriae-like adhesin FimI	*fimI*	Fimbriae-like adhesin FimI
fimC	Chaperone FimC	*fimC*	Chaperone FimC
fimD	Outer membrane usher protein SfmD	*fimD*	Outer membrane usher protein SfmD
fimH	Fimbriae-like adhesin SfmH	*fimH*	Fimbriae-like adhesin SfmH
fimF	Fimbriae-like periplasmic protein SfmF	*fimF*	Fimbriae-like periplasmic protein SfmF
fimZ	Transcriptional regulator of fimbriae expression FimZ	*fimZ*	Transcriptional regulator of fimbriae expression FimZ
fimY	Transcriptional regulator of fimbriae expression FimY	*fimY*	Transcriptional regulator of fimbriae expression FimY
fimW	Fimbriae W protein	*fimW*	Fimbriae W protein
csgC	Putative curli production protein CsgC	*csgC*	Putative curli production protein CsgC
csgA	Major curlin subunit precursor CsgA	*csgA*	Major curlin subunit precursor CsgA
csgB	Minor curlin subunit CsgB	*csgB*	Minor curlin subunit CsgB
csgD	Transcriptional regulator CsgD for 2nd curli operon	*csgD*	Transcriptional regulator CsgD for 2nd curli operon
csgE	Curli production assembly/transport component CsgE	*csgE*	Curli production assembly/transport component CsgE
csgF	Curli production assembly/transport component CsgF	*csgF*	Curli production assembly/transport component CsgF
csgG	Curli production assembly/transport component CsgG	*csgG*	Curli production assembly/transport component CsgG
SEE1_2078	PilV-like protein	*SEE2_2074*	PilV-like protein
SEE1_2079	Putative type IV pilin protein precursor	*SEE2_2075*	Putative type IV pilin protein precursor

SEE1_2080	Hypothetical protein	*SEE2_2076*	Hypothetical protein
SEE1_2081	Conjugal transfer protein TraA	*SEE2_2077*	Conjugal transfer protein TraA
pegD	Uncharacterized protein YehA precursor	*pegD*	Uncharacterized protein YehA precursor
pegC	Fimbriae usher protein StcC	*pegC*	Fimbriae usher protein StcC
pegB	Uncharacterized fimbrial chaperone YehC precursor	*pegB*	Uncharacterized fimbrial chaperone YehC precursor
pegA	Probable fimbrial chain protein stcA	*pegA*	Probable fimbrial chain protein stcA
SEE1_3011	Fimbriae usher protein StfC	*SEE2_3008*	Fimbriae usher protein StfC
SEE1_3012	Periplasmic fimbrial chaperone	*SEE2_3009*	Periplasmic fimbrial chaperone
SEE1_3013	MrfF	*SEE2_3010*	MrfF
SEE1_3014	MrfF	*SEE2_3011*	MrfF
SEE1_3015	Fimbrial subunit	*SEE2_3012*	Fimbrial subunit
stdC	Probable fimbrial chaperone protein	*stdC*	Probable fimbrial chaperone protein
stdB	FIG036507: Fimbriae usher protein StdB	*stdB*	FIG036507: Fimbriae usher protein StdB
stdA	Putative fimbrial-like protein	*stdA*	Putative fimbrial-like protein
hofQ	Type IV pilus biogenesis protein PilQ	*hofQ*	Type IV pilus biogenesis protein PilQ
yrfA	Type IV pilus biogenesis protein PilP	*yrfA*	Type IV pilus biogenesis protein PilP
yrfB	Type IV pilus biogenesis protein PilO	*yrfB*	Type IV pilus biogenesis protein PilO
yrfC	Type IV pilus biogenesis protein PilN	*yrfC*	Type IV pilus biogenesis protein PilN
yrfD	Type IV pilus biogenesis protein PilM	*yrfD*	Type IV pilus biogenesis protein PilM
lpfE	Putative fimbrial protein precursor	*lpfE*	Putative fimbrial protein precursor
lpfD	Putative fimbrial protein	*lpfD*	Putative fimbrial protein
lpfC	Type 1 fimbriae anchoring protein FimD	*lpfC*	Type 1 fimbriae anchoring protein FimD
lpfB	Chaperone protein lpfB precursor	*lpfB*	Chaperone protein lpfB precursor
lpfA	Long polar fimbria protein A precursor	*lpfA*	Long polar fimbria protein A precursor
SEE1_4547	Fimbrial protein precursor	*SEE2_4541*	Fimbrial protein precursor
SEE1_4548	Fimbrial chaperone protein	*SEE2_4542*	Fimbrial chaperone protein

SEE1_4549	Hypothetical protein	*SEE2_4543*	Hypothetical protein
SEE1_4550	Outer membrane fimbrial usher protein	*SEE2_4544*	Outer membrane fimbrial usher protein
sthE	Putative major fimbrial subunit	*sthE*	Putative major fimbrial subunit
sthD	Putative fimbrial subunit	*sthD*	Putative fimbrial subunit
sthB	Type 1 fimbriae anchoring protein FimD	*sthB*	Type 1 fimbriae anchoring protein FimD
sthA	Putative fimbrial chaperone protein	*sthA*	Putative fimbrial chaperone protein
SEE1_4673	Putative fimbrial protein	*SEE2_4667*	Putative fimbrial protein
pegD	Uncharacterized protein YehA precursor	*pegD*	Uncharacterized protein YehA precursor
pegC	Fimbriae usher protein StcC	*pegC*	Fimbriae usher protein StcC
pegB	Uncharacterized fimbrial chaperone YehC precursor	*pegB*	Uncharacterized fimbrial chaperone YehC precursor
pegA	Probable fimbrial chain protein stcA	*pegA*	Probable fimbrial chain protein stcA
ppdC	FIG004136: Prepilin peptidase dependent protein C precursor	*ppdC*	FIG004136: Prepilin peptidase dependent protein C precursor
ygdB	FIG006270: hypothetical protein	*ygdB*	FIG006270: hypothetical protein
ppdB	FIG004819: Prepilin peptidase dependent protein B precursor	*ppdB*	FIG004819: Prepilin peptidase dependent protein B precursor
ppdA	Prepilin peptidase dependent protein A precursor	*ppdA*	Prepilin peptidase dependent protein A precursor
stdC	Probable fimbrial chaperone protein	*stdC*	Probable fimbrial chaperone protein
stdB	FIG036507: Fimbriae usher protein StdB	*stdB*	FIG036507: Fimbriae usher protein StdB
stdA	Putative fimbrial-like protein	*stdA*	Putative fimbrial-like protein

Table S4. SEE1/SEE2 Non-Fimbrial Adhesin Genes

SEE1		SEE2	
**Gene**	**Description**	**Gene**	**Description**
SEE1_0229	Enhancin	*SEE2_0228*	Enhancin
yaeT	Outer membrane protein assembly factor YaeT	*yaeT*	Outer membrane protein assembly factor YaeT
hlpA	Outer membrane protein H precursor	*hlpA*	Outer membrane protein H precursor
SEE1_0348	Attachment invasion locus protein precursor	*SEE2_0347*	Attachment invasion locus protein precursor
ompX	Attachment invasion locus protein precursor	*ompX*	Attachment invasion locus protein precursor
ompF	Outer membrane protein F precursor	*ompF*	Outer membrane protein F precursor
ompA	Outer membrane protein A precursor	*ompA*	Outer membrane protein A precursor
ompC	Outer membrane protein C precursor	*ompC*	Outer membrane protein C precursor
ychP	Invasin	*ychP*	Invasin
ompW	Outer membrane protein W precursor	*ompW*	Outer membrane protein W precursor
SEE1_1469	Invasin-like protein	*SEE2_1464*	Invasin-like protein
SEE1_1638	Outer membrane protein C precursor	*SEE2_1631*	Outer membrane protein C precursor
ompN	Outer membrane protein N precursor	*ompN*	Outer membrane protein N precursor
pagC	Attachment invasion locus protein precursor	*pagC*	Attachment invasion locus protein precursor
ompC	Outer membrane protein C precursor	*ompC*	Outer membrane protein C precursor
shdA	AIDA autotransporter-like protein	*shdA*	AIDA autotransporter-like protein
sinH	Adherence and invasion outermembrane protein	*sinH*	Adherence and invasion outermembrane protein
yfgL	Outer membrane protein YfgL, lipoprotein component	*yfgL*	Outer membrane protein YfgL, lipoprotein component
SEE1_3097	Attachment invasion locus protein precursor	*SEE2_3094*	Attachment invasion locus protein precursor
yiaD	Outer membrane protein A precursor	*yiaD*	Outer membrane protein A precursor
misL	Autotransporter	*misL*	Autotransporter
yidE	Mediator of hyperadherence YidE	*yidE*	Mediator of hyperadherence YidE
yidQ	Outer membrane lipoprotein YidQ	*yidQ*	Outer membrane lipoprotein YidQ

Table S5. SEE1/SEE2 SPI-1 Related Genes

SEE1		SEE2	
**Gene**	**Description**	**Gene**	**Description**
avrA	Type III secretion injected virulence protein-NF-κB Inhibition	*avrA*	Type III secretion injected virulence protein-NF-κB Inhibition
sprB	SPI1-associated transcriptional regulator SprB	*sprB*	SPI1-associated transcriptional regulator SprB
hilC	Type III secretion transcriptional regulator HilC	*hilC*	Type III secretion transcriptional regulator HilC
SEE1_2918	Putative effector protein OrgC of SPI-1 type III secretion system	*SEE2_2915*	Putative effector protein OrgC of SPI-1 T3SS
ogrA	OrgB protein, associated with InvC ATPase of type III secretion system	*ogrA*	OrgB protein, associated with ATPase of T3SS
SEE1_2920	Oxygen-regulated invasion protein OrgA	*SEE2_2917*	Oxygen-regulated invasion protein OrgA
prgK	Type III secretion bridge between inner and outermembrane lipoprotein	*prgK*	Type III secretion bridge lipoprotein
prgJ	Type III secretion system protein	*prgJ*	Type III secretion system protein
prgI	Type III secretion cytoplasmic protein (YscF)	*prgI*	Type III secretion cytoplasmic protein (YscF)
prgH	Type III secretion protein EprH	*prgH*	Type III secretion protein EprH
hilD	Type III secretion transcriptional regulator HilD	*hilD*	Type III secretion transcriptional regulator HilD
hilA	Type III secretion transcriptional activator HilA	*hilA*	Type III secretion transcriptional activator HilA
iagB	Invasion protein IagB precursor	*iagB*	Invasion protein IagB precursor
sptP	Type III secretion injected virulence protein	*sptP*	Type III secretion injected virulence protein
sicP	secretion chaparone	*sicP*	secretion chaparone
SEE1_2930	Found within S. typhi pathogenicity island	*SEE2_2927*	Found within S. typhi pathogenicity island
iacP	Probable acyl carrier protein iacP	*iacP*	Probable acyl carrier protein iacP
sipA	Type III secretion injected virulence protein (YopE)	*sipA*	Type III secretion injected virulence protein (YopE)
sipD	Cell invasion protein SipD (Salmonella invasion protein D)	*sipD*	Cell invasion protein SipD (Salmonella invasion protein D)
sipC	Cell invasion protein sipC (Effector protein SipC)	*sipC*	Cell invasion protein sipC (Effector protein SipC)
sipB	Cell invasion protein SipB	*sipB*	Cell invasion protein SipB

sicA	Type III secretion chaperone protein for YopD (SycD)	*sicA*	Type III secretion chaperone protein for YopD (SycD)
spaS	Type III secretion inner membrane protein	*spaS*	Type III secretion inner membrane protein
spaR	Type III secretion inner membrane protein	*spaR*	Type III secretion inner membrane protein
spaQ	Type III secretion inner membrane protein	*spaQ*	Type III secretion inner membrane protein
spaP	Type III secretion inner membrane protein	*spaP*	Type III secretion inner membrane protein
spaO	Type III secretion inner membrane protein	*spaO*	Type III secretion inner membrane protein
invJ	Type III secretion host injection and negative regulator protein	*invJ*	Type III secretion host injection and negative regulator protein
invI	Surface presentation of antigens protein SpaM	*invI*	Surface presentation of antigens protein SpaM
invC	Probable ATP synthase SpaL (Invasion protein InvC)	*invC*	Probable ATP synthase SpaL (Invasion protein InvC)
invB	Type III secretion system protein BsaR	*invB*	Type III secretion system protein BsaR
invA	Type III secretion inner membrane channel protein	*invA*	Type III secretion inner membrane channel protein
invE	Type III secretion outermembrane contact sensing protein	*invE*	Type III secretion outermembrane contact sensing protein
invG	Type III secretion outermembrane pore forming protein	*invG*	Type III secretion outermembrane pore forming protein
invF	Type III secretion thermoregulatory protein	*invF*	Type III secretion thermoregulatory protein
invH	Invasion protein invH precursor	*invH*	Invasion protein invH precursor

Table S6. SEE1/SEE2 SPI-II Related Genes

SEE1		SEE2	
**Gene**	**Description**	**Gene**	**Description**
ssaU	Type III secretion inner membrane protein	*ssaU*	Type III secretion inner membrane protein
ssaT	Type III secretion inner membrane protein	*ssaT*	Type III secretion inner membrane protein
ssaS	Type III secretion inner membrane protein	*ssaS*	Type III secretion inner membrane protein
ssaR	Type III secretion inner membrane protein	*ssaR*	Type III secretion inner membrane protein
ssaQ	Type III secretion inner membrane protein	*ssaQ*	Type III secretion inner membrane protein
ssaP	Type III secretion protein (YscP)	*ssaP*	Type III secretion protein (YscP)
ssaO	Type III secretion spans bacterial envelope protein (YscO)	*ssaO*	Type III secretion spans bacterial envelope protein (YscO)
ssaN	Flagellum-specific ATP synthase FliI	*ssaN*	Flagellum-specific ATP synthase FliI
ssaV	Type III secretion inner membrane channel protein	*ssaV*	Type III secretion inner membrane channel protein
ssaM	Secretion system apparatus protein SsaM	*ssaM*	Secretion system apparatus protein SsaM
ssaL	Type III secretion cytoplasmic protein (YscL)	*ssaL*	Type III secretion cytoplasmic protein (YscL)
ssaK	Type III secretion protein SsaK	*ssaK*	Type III secretion protein SsaK
SEE1_1759	FIG029138: Type III secretion protein	*SEE2_1752*	FIG029138: Type III secretion protein
ssaJ	Type III secretion bridge between IM and OM	*ssaJ*	Type III secretion bridge between IM and OM
ssaI	Type III secretion protein SsaI	*ssaI*	Type III secretion protein SsaI
ssaH	Type III secretion protein SsaH	*ssaH*	Type III secretion protein SsaH
ssaG	Type III secretion protein SsaG	*ssaG*	Type III secretion protein SsaG
sseG	Secretion system effector SseG	*sseG*	Secretion system effector SseG
sseF	Type III secretion effector SseF	*sseF*	Type III secretion effector SseF
sscB	Secretion system chaparone SscB	*sscB*	Secretion system chaparone SscB
sseE	Secretion system effector SseE	*sseE*	Secretion system effector SseE
sseD	Secretion system effector SseD	*sseD*	Secretion system effector SseD
sseC	Secretion system effector SseC	*sseC*	Secretion system effector SseC
sscA	Secretion system chaparone SscA	*sscA*	Secretion system chaparone SscA
sseB	Secretion system effector SseB	*sseB*	Secretion system effector SseB

sseA	Type III secretion system chaperone SseA	*sseA*	Type III secretion system chaperone SseA
ssaE	Secretion system effector SsaE	*ssaE*	Secretion system effector SsaE
ssaD	Secretion system apparatus SsaD	*ssaD*	Secretion system apparatus SsaD
ssaC	Type III secretion outermembrane pore forming protein	*ssaC*	Type III secretion outermembrane pore forming protein
ssaB	Type III secretion system effector protein	*ssaB*	Type III secretion system effector protein
ssrA	Secretion system regulator: Sensor component	*ssrA*	Secretion system regulator: Sensor component
ssrB	Secretion system regulator of DegU/UvrY/BvgA type	*ssrB*	Secretion system regulator of DegU/UvrY/BvgA type
orf242	Transcriptional regulator associated with photolyase	*orf242*	Transcriptional regulator associated with photolyase
orf319	COG1683: Uncharacterized conserved protein	*orf319*	COG1683: Uncharacterized conserved protein
orf70	FIG01045422: hypothetical protein	*orf70*	FIG01045422: hypothetical protein

Table S7. SEE1/SEE2 SPI-III Related Genes

SEE1		SEE2	
**Gene**	**Description**	**Gene**	**Description**
SEE1_3848	FIG01046146: hypothetical protein	*SEE2_3841*	FIG01046146: hypothetical protein
SEE1_3849	Putative DNA-binding protein in cluster with Type I RMS	*SEE2_3842*	Putative DNA-binding protein in cluster with Type I RMS
SEE1_3850	FIG01046502: hypothetical protein	*SEE2_3843*	FIG01046502: hypothetical protein
rmbA	RmbA	*rmbA*	RmbA
misL	autotransporter	*misL*	autotransporter
fidL	YqeJ protein	*fidL*	YqeJ protein
marT	Putative sensory transducer	*marT*	Putative sensory transducer
SEE1_3855	FIG01046505: hypothetical protein	*SEE2_3848*	FIG01046505: hypothetical protein
slsA	Nicotinamidase family protein YcaC	*slsA*	Nicotinamidase family protein YcaC
cigR	Putative inner membrane protein	*cigR*	Putative inner membrane protein
mgtB	Mg(2+) transport ATPase, P-type (EC 3.6.3.2)	*mgtB*	Mg(2+) transport ATPase, P-type (EC 3.6.3.2)
SEE1_3859	FIG01045269: hypothetical protein	*SEE2_3852*	FIG01045269: hypothetical protein
mgtC	Mg(2+) transport ATPase protein C	*mgtC*	Mg(2+) transport ATPase protein C

Table S8. SEE1/SEE2 SPI-IV Related Genes

SEE1		SEE2	
**Gene**	**Description**	**Gene**	**Description**
SEE1_4324	Putative inner membrane protein or exported protein SiiA	*SEE2_4317*	Putative inner membrane protein or exported protein SiiA
SEE1_4325	Putative integral membrane protein SiiB	*SEE2_4318*	Putative integral membrane protein SiiB
SEE1_4326	Agglutination protein SiiC	*SEE2_4319*	Agglutination protein SiiC
SEE1_4327	Putative type-I secretion protein SiiD	*SEE2_4320*	Putative type-I secretion protein SiiD
SEE1_4328	Large repetitive protein SiiE	*SEE2_4321*	Large repetitive protein SiiE
SEE1_4329	Putative type-1 secretion protein SiiF	*SEE2_4322*	Putative type-1 secretion protein SiiF
yjcB	YjcB protein	*yjcB*	YjcB protein
yjcC	FIG00638940: hypothetical protein	*yjcC*	FIG00638940: hypothetical protein
soxS	Regulatory protein SoxS	*soxS*	Regulatory protein SoxS
soxR	Redox-sensitive transcriptional activator SoxR	*soxR*	Redox-sensitive transcriptional activator SoxR

Table S9. SEE1/SEE2 SPI-V Related Genes

SEE1		SEE2	
**Gene**	**Description**	**Gene**	**Description**
pipA	Pathogenicity island encoded protein: SPI3	*pipA*	Pathogenicity island encoded protein: SPI3
pipB	FIG01046201: hypothetical protein	*pipB*	FIG01046201: hypothetical protein
pipC	Invasion gene E protein	*SEE2_1016*	Hypothetical Protein
sopB	Inositol phosphate phosphatase sopB (EC 3.1.3.-)	*pipC*	Invasion gene E protein (Pathogenicity island encoded protein)
pipD	Probable dipeptidase (EC 3.4.-.-)	*sopB*	Inositol phosphate phosphatase sopB (EC 3.1.3.-)
copR	Putative two component system histidine kinase YedV	*SEE2_1019*	FIG01045843: hypothetical protein
copS	Putative two-component system response regulator YedW	*pipD*	Probable dipeptidase (EC 3.4.-.-)
		copR	Putative two component system histidine kinase YedV
		copS	Putative two-component system response regulator YedW

Table S10. SEE1/SEE2 Non-SPI Related Toxins and Secretion System Genes

SEE1		SEE2	
**Gene**	**Description**	**Gene**	**Description**
lpxD	UDP-3-O-[3-hydroxymyristoyl] glucosamine N-acyltransferase	*lpxD*	UDP-3-O-[3-hydroxymyristoyl] glucosamine N-acyltransferase
fabZ	(3R)-hydroxymyristoyl-[acyl carrier protein] dehydratase	*fabZ*	(3R)-hydroxymyristoyl-[acyl carrier protein] dehydratase
lpxA	Acyl-[acyl-carrier-protein]--UDP-N-acetylglucosamine O-acyltransferase	*lpxA*	Acyl-[acyl-carrier-protein]--UDP-N-acetylglucosamine O-acyltransferase
lpxB	Lipid-A-disaccharide synthase	*lpxB*	Lipid-A-disaccharide synthase
SEE1_0280	Uncharacterized protein ImpA	*SEE2_0279*	Uncharacterized protein ImpA
SEE1_0281	Uncharacterized protein ImpH/VasB	*SEE2_0280*	Uncharacterized protein ImpH/VasB
SEE1_0282	Probable secreted protein	*SEE2_0281*	Probable secreted protein
SEE1_0283	Putative cytoplasmic protein	*SEE2_0282*	Putative cytoplasmic protein
SEE1_0284	VgrG protein	*SEE2_0283*	VgrG protein
ybjX	Virulence factor VirK	*ybjX*	Virulence factor VirK
SEE1_0937	FIG01046987: hypothetical protein	*SEE2_0935*	FIG01046987: hypothetical protein
himD	Integration host factor beta subunit	*himD*	Integration host factor beta subunit
SEE1_0986	Virulence protein msgA	*SEE2_0983*	Virulence protein msgA
SEE1_1074	Secreted protein Hcp	*SEE2_1070*	Secreted protein Hcp
SEE1_1075	IcmF-related protein	*SEE2_1072*	IcmF-related protein
SEE1_1229	GtgA	*SEE2_1224*	GtgA
msbB	Lipid A biosynthesis (KDO) 2-(lauroyl)-lipid IVA acyltransferase	*msbB*	Lipid A biosynthesis (KDO) 2-(lauroyl)-lipid IVA acyltransferase
SEE1_1241	Invasion-associated secreted protein.	*SEE2_1236*	Invasion-associated secreted protein.
sopE2	G-nucleotide exchange factor SopE	*sopE2*	G-nucleotide exchange factor SopE
sseJ	Secreted effector J SseJ Deacylase	*sseJ*	Secreted effector J SseJ Deacelase
ydcP	Putative collagenase	*ydcP*	Putative collagenase
sifB	Secreted effector protein	*sifB*	Secreted effector protein
srfC	Putative virulence factor	*srfC*	Putative virulence factor
srfB	SrfB	*srfB*	SrfB

srfA	Putative virulence effector protein	*srfA*	Putative virulence effector protein
sppA	Protease IV	*sppA*	Protease IV
himA	Integration host factor alpha subunit	*himA*	Integration host factor alpha subunit
rfc	O-antigen polymerase	*rfc*	O-antigen polymerase
pagD	Putative outer membrane virulence protein	*pagD*	Putative outer membrane virulence protein
envE	Probable lipoprotein envE precursor	*envE*	Probable lipoprotein envE precursor
msgA	Virulence protein MsgA	*msgA*	Virulence protein MsgA
envF	Probable lipoprotein envF precursor	*envF*	Probable lipoprotein envF precursor
sifA	SifA protein	*sifA*	SifA protein
mviN	Proposed peptidoglycan lipid II flippase MurJ	*mviN*	Proposed peptidoglycan lipid II flippase MurJ
mviM	Virulence factor MviM	*mviM*	Virulence factor MviM
htrB	Lipid A biosynthesis lauroyl acyltransferase	*htrB*	Lipid A biosynthesis lauroyl acyltransferase
sopA	Secreted effector protein	*sopA*	Secreted effector protein
wzzB	Regulator of length of O-antigen component of LPS chains	*wzzB*	Regulator of length of O-antigen component of LPS chains
udg	UDP-glucose dehydrogenase	*udg*	UDP-glucose dehydrogenase
gnd	6-phosphogluconate dehydrogenase, decarboxylating	*gnd*	6-phosphogluconate dehydrogenase, decarboxylating
rfbP	Undecaprenyl-phosphate galactosephosphotransferase	*rfbP*	Undecaprenyl-phosphate galactosephosphotransferase
rfbK	Phosphomannomutase	*rfbK*	Phosphomannomutase
rfbM	Mannose-1-phosphate guanylyltransferase (GDP)	*rfbM*	Mannose-1-phosphate guanylyltransferase (GDP)
rfbN	O antigen biosynthesis rhamnosyltransferase rfbN	*rfbN*	O antigen biosynthesis rhamnosyltransferase rfbN
rfbU	O-antigen flippase Wzx	*rfbU*	O-antigen flippase Wzx
rfbV	Putative glycosyltransferase	*rfbV*	Putative glycosyltransferase
rfbX	O-antigen flippase Wzx	*rfbX*	O-antigen flippase Wzx
rfbE	dTDP-glucose 4,6-dehydratase	*rfbE*	dTDP-glucose 4,6-dehydratase
rfbS	UDP-glucose 4-epimerase	*rfbS*	UDP-glucose 4-epimerase
rfbH	CDP-4-dehydro-6-deoxy-D-glucose 3-dehydratase	*rfbH*	CDP-4-dehydro-6-deoxy-D-glucose 3-dehydratase
rfbG	Similar to CDP-glucose 4,6-dehydratase	*rfbG*	Similar to CDP-glucose 4,6-dehydratase

rfbF	Glucose-1-phosphate cytidylyltransferase	*rfbF*	Glucose-1-phosphate cytidylyltransferase
rfbI	CDP-6-deoxy-delta-3,4-glucoseen reductase-like	*rfbI*	CDP-6-deoxy-delta-3,4-glucoseen reductase-like
rfbC	dTDP-4-dehydrorhamnose 3,5-epimerase	*rfbC*	dTDP-4-dehydrorhamnose 3,5-epimerase
rfbA	Glucose-1-phosphate thymidylyltransferase	*rfbA*	Glucose-1-phosphate thymidylyltransferase
rfbD	dTDP-4-dehydrorhamnose reductase	*rfbD*	dTDP-4-dehydrorhamnose reductase
rfbB	dTDP-glucose 4,6-dehydratase	*rfbB*	dTDP-glucose 4,6-dehydratase
sspH2	Secreted effector protein-Inhibit Actin Polymerization	*sspH2*	Secreted effector proteinInhibit Actin Polymerization
wzc	Tyrosine-protein kinase Wzc	*wzc*	Tyrosine-protein kinase Wzc
wzb	Low molecular weight protein-tyrosine-phosphatase Wzb	*wzb*	Low molecular weight protein-tyrosine-phosphatase Wzb
wza	Polysaccharide export lipoprotein Wza	*wza*	Polysaccharide export lipoprotein Wza
SEE1_2350	Homolog of virulence protein msgA	*SEE2_2348*	Homolog of virulence protein msgA
yfbK	Von Willebrand factor type A domain protein	*yfbK*	Von Willebrand factor type A domain protein
pgtE	Protease VII (Omptin) precursor	*pgtE*	Protease VII (Omptin) precursor
ddg	Lipid A biosynthesis lauroyl acyltransferase	*ddg*	Lipid A biosynthesis lauroyl acyltransferase
yfjD	Hemolysins and related proteins containing CBS domains	*yfjD*	Hemolysins and related proteins containing CBS domains
SEE1_2759	Large repetitive protein	*SEE2_2755*	Large repetitive protein
SEE1_2760	FIG01045638: hypothetical protein	*SEE2_2756*	FIG01045638: hypothetical protein
SEE1_2761	Putative type I secretion protein, ATP-binding protein	*SEE2_2757*	Putative type I secretion protein, ATP-binding protein
SEE1_2762	Putative HlyD family secretion protein	*SEE2_2758*	Putative HlyD family secretion protein
SEE1_2822	Similar to pipB	*SEE2_2819*	Similar to pipB
virK	Virulence protein VirK	*virK*	Virulence protein VirK
sopD	Secreted protein	*yfjD*	Secreted protein
SEE1_3099	VapC toxin protein	*SEE2_3096*	VapC toxin protein
yqfA	COG1272: Predicted membrane protein hemolysin III homolog	*yqfA*	COG1272: Predicted membrane protein hemolysin III homolog
yqfB	Protein HI1394	*yqfB*	Protein HI1394

tolC	Type I secretion outer membrane protein, TolC precursor	*tolC*	Type I secretion outer membrane protein, TolC precursor
yraP	21 kDa hemolysin precursor	*yraP*	21 kDa hemolysin precursor
bigA	Putative surface-exposed virulence protein	*bigA*	Putative surface-exposed virulence protein
SEE1_3595	YafQ toxin protein	*SEE2_3589*	YafQ toxin protein
rfaD	ADP-L-glycero-D-manno-heptose-6-epimerase	*rfaD*	ADP-L-glycero-D-manno-heptose-6-epimerase
rfaF	ADP-heptose--lipooligosaccharide heptosyltransferase II	*rfaF*	ADP-heptose--lipooligosaccharide heptosyltransferase II
rfaC	LPS heptosyltransferase I	*rfaC*	LPS heptosyltransferase I
rfaL	Oligosaccharide repeat unit polymerase Wzy; O-antigen ligase	*rfaL*	Oligosaccharide repeat unit polymerase Wzy; O-antigen ligase
rfaK	LPS 1,2-N-acetylglucosaminetransferase	*rfaK*	LPS 1,2-N-acetylglucosaminetransferase
rfaZ	LPS core biosynthesis protein RfaZ	*rfaZ*	LPS core biosynthesis protein RfaZ
rfaY	LPS core biosynthesis protein RfaY	*rfaY*	LPS core biosynthesis protein RfaY
rfaJ	UDP-glucose:(glucosyl)LPS alpha-1,2-glucosyltransferase	*rfaJ*	UDP-glucose:(glucosyl)LPS alpha-1,2-glucosyltransferase
rfaI	UDP-glucose:(glucosyl)LPS alpha-1,3-glucosyltransferase	*rfaI*	UDP-glucose:(glucosyl)LPS alpha-1,3-glucosyltransferase
rfaB	LPS 1,6-galactosyltransferase	*rfaB*	LPS 1,6-galactosyltransferase
rfaP	LPS core biosynthesis protein WaaP, heptosyl-I-kinase	*rfaP*	LPS core biosynthesis protein WaaP, heptosyl-I-kinase
rfaG	UDP-glucose:(heptosyl) LPS alpha1,3-glucosyltransferase WaaG	*rfaG*	UDP-glucose:(heptosyl) LPS alpha1,3-glucosyltransferase WaaG
rfaQ	LPS heptosyltransferase III	*rfaQ*	LPS heptosyltransferase III
wzzE	Regulator of length of O-antigen component of LPS chains	*wzzE*	Regulator of length of O-antigen component of LPS chains
wecB	UDP-N-acetylglucosamine 2-epimerase	*wecB*	UDP-N-acetylglucosamine 2-epimerase
wecC	UDP-glucose dehydrogenase	*wecC*	UDP-glucose dehydrogenase
rffG	dTDP-glucose 4,6-dehydratase	*rffG*	dTDP-glucose 4,6-dehydratase
SEE1_4006	Glucose-1-phosphate thymidylyltransferase	*SEE2_3999*	Glucose-1-phosphate thymidylyltransferase
rffC	LPS biosynthesis protein RffC	*rffC*	LPS biosynthesis protein RffC

wecE	4-keto-6-deoxy-N-Acetyl-D-hexosaminyl-(Lipid carrier) aminotransferase	*wecE*	4-keto-6-deoxy-N-Acetyl-D-hexosaminyl-(Lipid carrier) aminotransferase
wzxE	WzxE protein	*wzxE*	WzxE protein
virF	Virulence regulon transcriptional activator virF	*virF*	Virulence regulon transcriptional activator virF
SEE1_4010	4-alpha-L-fucosyltransferase (EC 2.4.1.-)	*SEE2_4003*	4-alpha-L-fucosyltransferase (EC 2.4.1.-)
wecF	Putative ECA polymerase	*wecF*	Putative ECA polymerase
wecG	Probable UDP-N-acetyl-D-mannosaminuronic acid transferase	*wecG*	Probable UDP-N-acetyl-D-mannosaminuronic acid transferase
SEE1_0285	Rhs-family protein	*SEE2_0284*	Rhs-family protein
SEE1_0289	rhs core protein with extension	*SEE2_0288*	rhs core protein with extension
sseI	E3 Ubiquitin Ligase	sseI	E3 Ubiquitin Ligase

Table S11. SEE1/SEE2 Iron Sequestration Genes

SEE1		SEE2	
**Gene**	**Description**	**Gene**	**Description**
fhuA	Ferric hydroxamate outer membrane receptor FhuA	*fhuA*	Ferric hydroxamate outer membrane receptor FhuA
fhuC	Ferric hydroxamate ABC transporter, ATP-binding protein FhuC	*fhuC*	Ferric hydroxamate ABC transporter, ATP-binding protein FhuC
fhuD	Ferric hydroxamate ABC transporter, periplasmic substrate binding protein FhuD	*fhuD*	Ferric hydroxamate ABC transporter, periplasmic substrate binding protein FhuD
fhuB	Ferric hydroxamate ABC transporter, permease component FhuB	*fhuB*	Ferric hydroxamate ABC transporter, permease component FhuB
foxA	Ferrichrome-iron receptor	*foxA*	Ferrichrome-iron receptor
entD	4'-phosphopantetheinyl transferase/[enterobactin] siderophore	*entD*	4'-phosphopantetheinyl transferase/[enterobactin] siderophore
fepA	TonB-dependent receptor; Outer membrane receptor for ferric enterobactin	*fepA*	TonB-dependent receptor; Outer membrane receptor for ferric enterobactin
fes	Enterobactin esterase	*fes*	Enterobactin esterase
ybdZ	FIG005032: Putative cytoplasmic protein YbdZ in enterobactin biosynthesis operon	*ybdZ*	FIG005032: Putative cytoplasmic protein YbdZ in enterobactin biosynthesis operon
entF	Enterobactin synthetase component F, serine activating enzyme	*entF*	Enterobactin synthetase component F, serine activating enzyme
fepE	Ferric enterobactin uptake protein FepE	*fepE*	Ferric enterobactin uptake protein FepE
fepC	Ferric enterobactin transport ATP-binding protein FepC	*fepC*	Ferric enterobactin transport ATP-binding protein FepC
fepG	Ferric enterobactin transport system permease protein FepG	*fepG*	Ferric enterobactin transport system permease protein FepG
fepD	Ferric enterobactin transport system permease protein FepD	*fepD*	Ferric enterobactin transport system permease protein FepD
entS	Enterobactin exporter EntS	*entS*	Enterobactin exporter EntS
fepB	Ferric enterobactin-binding periplasmic protein FepB	*fepB*	Ferric enterobactin-binding periplasmic protein FepB
entC	Isochorismate synthase/enterobactin] siderophore	*entC*	Isochorismate synthase/enterobactin] siderophore
entE	2,3-dihydroxybenzoate-AMP ligase/[enterobactin] siderophore	*entE*	2,3-dihydroxybenzoate-AMP ligase/[enterobactin] siderophore
entB	Isochorismatase/[enterobactin] siderophore/Apo-aryl carrier domain of EntB	*entB*	Isochorismatase/[enterobactin] siderophore/Apo-aryl carrier domain of EntB
entA	2,3-dihydro-2,3-dihydroxybenzoate dehydrogenase/[enterobactin] siderophore	*entA*	2,3-dihydro-2,3-dihydroxybenzoate dehydrogenase/[enterobactin] siderophore

ybdB	Proofreading thioesterase in enterobactin biosynthesis EntH	*ybdB*	Proofreading thioesterase in enterobactin biosynthesis EntH
fur	Ferric uptake regulation protein FUR	*fur*	Ferric uptake regulation protein FUR
ftn	Ferritin-like protein 2	*ftn*	Ferritin-like protein 2
ftnB	Ferritin-like protein 2	*ftnB*	Ferritin-like protein 2
tonB	Ferric siderophore transport system, periplasmic protein TonB	*tonB*	Ferric siderophore transport system, periplasmic binding protein TonB
fhuE	Putative OMR family iron-siderophore receptor precursor	*fhuE*	Putative OMR family iron-siderophore receptor precursor
iroB	Glycosyltransferase IroB	*iroB*	Glycosyltransferase IroB
iroC	ABC transporter protein IroC	*iroC*	ABC transporter protein IroC
iroD	Trilactone hydrolase IroD	*iroD*	Trilactone hydrolase IroD
iroE	Periplasmic esterase IroE	*iroE*	Periplasmic esterase IroE
iroN	Outer Membrane Siderophore Receptor IroN	*iroN*	Outer Membrane Siderophore Receptor IroN
btuB	Outer membrane vitamin B12 receptor BtuB	*yqjH*	Outer membrane vitamin B12 receptor BtuB
yqjH	iron-chelator utilization protein	*bfr*	iron-chelator utilization protein
bfr	Bacterioferritin	*feoA*	Bacterioferritin
feoA	Ferrous iron transport protein A	*feoB*	Ferrous iron transport protein A
feoB	Ferrous iron transport protein B	*yhgH*	Ferrous iron transport protein B
yhgH	Ferrous iron-sensisng transcriptional regulator FeoC	*btuB*	Ferrous iron-sensisng transcriptional regulator FeoC

Table S12. SEE1/SEE2 Signaling and Motility Genes

SEE1		SEE2	
**Gene**	**Description**	**Gene**	**Description**
yaiU	Putative flagellin structural protein	*yaiU*	Putative flagellin structural protein
fliR	Flagellar biosynthesis protein FliR	*fliR*	Flagellar biosynthesis protein FliR
fliQ	Flagellar biosynthesis protein FliQ	*fliQ*	Flagellar biosynthesis protein FliQ
fliP	Flagellar biosynthesis protein FliP	*fliP*	Flagellar biosynthesis protein FliP
fliO	Flagellar biosynthesis protein FliQ	*fliO*	Flagellar biosynthesis protein FliQ
fliN	Flagellar motor switch protein FliN	*fliN*	Flagellar motor switch protein FliN
fliM	Flagellar motor switch protein FliM	*fliM*	Flagellar motor switch protein FliM
fliL	Flagellar biosynthesis protein FliL	*fliL*	Flagellar biosynthesis protein FliL
fliK	Flagellar hook-length control protein FliK	*fliK*	Flagellar hook-length control protein FliK
fliJ	Flagellar protein FliJ	*fliJ*	Flagellar protein FliJ
fliI	Flagellum-specific ATP synthase FliI	*fliI*	Flagellum-specific ATP synthase FliI
fliH	Flagellar assembly protein FliH	*fliH*	Flagellar assembly protein FliH
fliG	Flagellar motor switch protein FliG	*fliG*	Flagellar motor switch protein FliG
fliF	Flagellar M-ring protein FliF	*fliF*	Flagellar M-ring protein FliF
fliE	Flagellar hook-basal body complex protein FliE	*fliE*	Flagellar hook-basal body complex protein FliE
yedF	UPF0033 protein YedF	*yedF*	UPF0033 protein YedF
yedE	Putative transport system permease protein	*yedE*	Putative transport system permease protein
fliT	Flagellar biosynthesis protein FliT	*fliT*	Flagellar biosynthesis protein FliT
fliS	Flagellar biosynthesis protein FliS	*fliS*	Flagellar biosynthesis protein FliS
fliD	Flagellar hook-associated protein FliD	*fliD*	Flagellar hook-associated protein FliD
fljB	Flagellar biosynthesis protein FliC	*fljB*	Flagellar biosynthesis protein FliC
fliB	Lysine-N-methylase	*fliB*	Lysine-N-methylase
fliA	RNA polymerase sigma factor for flagellar operon	*fliA*	RNA polymerase sigma factor for flagellar operon

fliZ	Flagellar biosynthesis protein FliZ	*fliZ*	Flagellar biosynthesis protein FliZ
fliY	Cystine ABC transporter, periplasmic cystine-binding protein FliY	*fliY*	Cystine ABC transporter, periplasmic cystine-binding protein FliY
flhD	Flagellar transcriptional activator FlhD	*flhD*	Flagellar transcriptional activator FlhD
flhC	Flagellar transcriptional activator FlhC	*flhC*	Flagellar transcriptional activator FlhC
motA	Flagellar motor rotation protein MotA	*motA*	Flagellar motor rotation protein MotA
motB	Flagellar motor rotation protein MotB	*motB*	Flagellar motor rotation protein MotB
cheA	Signal transduction histidine kinase CheA	*cheA*	Signal transduction histidine kinase CheA
cheW	Positive regulator of CheA protein activity (CheW)	*cheW*	Positive regulator of CheA protein activity (CheW)
cheM	Methyl-accepting chemotaxis protein II	*cheM*	Methyl-accepting chemotaxis protein II
cheR	Chemotaxis protein methyltransferase CheR	*cheR*	Chemotaxis protein methyltransferase CheR
cheB	Chemotaxis response regulator protein-glutamate methylesterase CheB	*cheB*	Chemotaxis response regulator protein-glutamate methylesterase CheB
cheY	Chemotaxis regulator - transmits chemoreceptor signals to CheY	*cheY*	Chemotaxis regulator - transmits chemoreceptor signals to CheY
cheZ	Chemotaxis response - phosphatase CheZ	*cheZ*	Chemotaxis response - phosphatase CheZ
flhB	Flagellar biosynthesis protein FlhB	*flhB*	Flagellar biosynthesis protein FlhB
flhA	Flagellar biosynthesis protein FlhA	*flhA*	Flagellar biosynthesis protein FlhA
flhE	Flagellar protein FlhE	*flhE*	Flagellar protein FlhE
trg	Methyl-accepting chemotaxis protein III	*trg*	Methyl-accepting chemotaxis protein III
flgL	Flagellar hook-associated protein FlgL	*flgL*	Flagellar hook-associated protein FlgL
flgK	Flagellar hook-associated protein FlgK	*flgK*	Flagellar hook-associated protein FlgK
flgJ	Flagellar protein FlgJ [peptidoglycan hydrolase]	*flgJ*	Flagellar protein FlgJ [peptidoglycan hydrolase]
flgI	Flagellar P-ring protein FlgI	*flgI*	Flagellar P-ring protein FlgI
flgH	Flagellar L-ring protein FlgH	*flgH*	Flagellar L-ring protein FlgH
flgG	Flagellar basal-body rod protein FlgG	*flgG*	Flagellar basal-body rod protein FlgG

flgF	Flagellar basal-body rod protein FlgF	*flgF*	Flagellar basal-body rod protein FlgF
flgE	Flagellar hook protein FlgE	*flgE*	Flagellar hook protein FlgE
flgD	Flagellar basal-body rod modification protein FlgD	*flgD*	Flagellar basal-body rod modification protein FlgD
flgC	Flagellar basal-body rod protein FlgC	*flgC*	Flagellar basal-body rod protein FlgC
flgB	Flagellar basal-body rod protein FlgB	*flgB*	Flagellar basal-body rod protein FlgB
flgA	Flagellar basal-body P-ring formation protein FlgA	*flgA*	Flagellar basal-body P-ring formation protein FlgA
flgM	Negative regulator of flagellin synthesis FlgM	*flgM*	Negative regulator of flagellin synthesis FlgM
flgN	Flagellar biosynthesis protein FlgN	*flgN*	Flagellar biosynthesis protein FlgN
cheV	Chemotaxis protein CheV	*cheV*	Chemotaxis protein CheV
yggR	Twitching motility protein PilT	*yggR*	Twitching motility protein PilT
SEE1_3213	Putative methyl-accepting chemotaxis protein	*SEE2_3755*	Putative methyl-accepting chemotaxis protein
SEE1_3228	Methyl-accepting chemotaxis protein I	*SEE2_4375*	Methyl-accepting chemotaxis protein I
SEE1_3761	Putative chemotaxis protein, resembles cheA	*SEE2_4376*	Putative chemotaxis protein, resembles cheA
tsr	Methyl-accepting chemotaxis protein I	*tsr*	Methyl-accepting chemotaxis protein I
SEE1_4382	Flagellar regulon repressor RtsB	*SEE2_3209*	Flagellar regulon repressor RtsB
SEE1_4383	Type III secretion and flagellar regulator RtsA	*SEE2_3224*	Type III secretion and flagellar regulator RtsA
phoB	Phosphate regulon transcriptional regulatory protein PhoB	*phoB*	Phosphate regulon transcriptional regulatory protein PhoB
phoR	Phosphate regulon sensor protein PhoR	*phoR*	Phosphate regulon sensor protein PhoR
copR	Putative two component system histidine kinase YedV	*copR*	Putative two component system histidine kinase YedV
copS	Putative two-component system response regulator YedW	*copS*	Putative two-component system response regulator YedW
sdiA	N-3-oxohexanoyl-L-homoserine lactone quorum-sensing transcriptional activator	*sdiA*	N-3-oxohexanoyl-L-homoserine lactone quorum-sensing transcriptional activator
SEE1_0172	Cellular communication/signal transduction	*SEE2_0171*	Cellular communication/signal transduction
ynaI	Mechanosensitive ion channel	*ynaI*	Mechanosensitive ion channel

ynaJ	Putative inner membrane protein	*ynaJ*	Putative inner membrane protein
trg	Methyl-accepting chemotaxis protein III	*trg*	Methyl-accepting chemotaxis protein III
ydcI	LysR family transcriptional regulator YdcI	*ydcI*	LysR family transcriptional regulator YdcI
rstA	Transcriptional regulatory protein RstA	*rstA*	Transcriptional regulatory protein RstA
rstB	Sensory histidine kinase two-component regulatory system with RstA	*rstB*	Sensory histidine kinase two-component regulatory system with RstA
phoP	Transcriptional regulatory protein PhoP	*phoP*	Transcriptional regulatory protein PhoP
phoQ	Sensor protein PhoQ	*phoQ*	Sensor protein PhoQ
baeS	Sensory histidine kinase BaeS	*baeS*	Sensory histidine kinase BaeS
baeR	Response regulator BaeR	*baeR*	Response regulator BaeR
yehT	Hypothetical response regulatory protein yehT	*yehT*	Hypothetical response regulatory protein yehT
yehU	Autolysin sensor kinase	*yehU*	Autolysin sensor kinase
yojN	Two-component sensor protein RcsD	*yojN*	Two-component sensor protein RcsD
rcsC	Two-component sensor protein RcsC	*rcsB*	Two-component sensor protein RcsC
rcsB	DNA-binding capsular synthesis response regulator RcsB	*rcsC*	DNA-binding capsular synthesis response regulator RcsB
luxS	S-ribosylhomocysteine lyase/Autoinducer-2 production protein LuxS	*luxS*	S-ribosylhomocysteine lyase/Autoinducer-2 production protein LuxS
barA	BarA sensory histidine kinase	*barA*	BarA sensory histidine kinase
ygiX	Two-component system response regulator QseB	*ygiX*	Two-component system response regulator QseB
ygiY	Sensory histidine kinase QseC	*ygiY*	Sensory histidine kinase QseC
SEE1_3296	Methyl-accepting chemotaxis protein I	*SEE2_3291*	Methyl-accepting chemotaxis protein I
aer	Aerotaxis sensor receptor protein	*aer*	Aerotaxis sensor receptor protein
mscL	Large-conductance mechanosensitive channel	*mscL*	Large-conductance mechanosensitive channel
envZ	Osmolarity sensory histidine kinase EnvZ	*envZ*	Osmolarity sensory histidine kinase EnvZ
ompR	Two-component system response regulator OmpR	*ompR*	Two-component system response regulator OmpR
rthB	Homoserine/homoserine lactone efflux protein	*rthB*	Homoserine/homoserine lactone efflux protein

aer	Aerotaxis sensor receptor protein	*aer*	Aerotaxis sensor receptor protein
ego	Autoinducer 2 (AI-2) ABC transport system,ATP-binding component	*ego*	Autoinducer 2 (AI-2) ABC transport system,ATP-binding component
ydeY	Autoinducer 2 (AI-2) ABC transport system, membrane channel protein LsrC	*ydeY*	Autoinducer 2 (AI-2) ABC transport system, membrane channel protein LsrC
ydeZ	Autoinducer 2 (AI-2) ABC transport system, membrane channel protein LsrD	*ydeZ*	Autoinducer 2 (AI-2) ABC transport system, membrane channel protein LsrD
yneA	Autoinducer 2 (AI-2) ABC transport system, periplasmic AI-2 binding protein LsrB	*yneA*	Autoinducer 2 (AI-2) ABC transport system, periplasmic AI-2 binding protein LsrB
yneB	Autoinducer 2 (AI-2) aldolase LsrF	*yneB*	Autoinducer 2 (AI-2) aldolase LsrF
yneC	Autoinducer 2 (AI-2) modifying protein LsrG	*yneC*	Autoinducer 2 (AI-2) modifying protein LsrG
encR	Putative luxR family bacterial regulatory protein	*encR*	Putative luxR family bacterial regulatory protein
ydeV	Autoinducer 2 (AI-2) kinase LsrK	*ydeV*	Autoinducer 2 (AI-2) kinase LsrK
ydeW	LsrR, transcriptional repressor of lsr operon	*ydeW*	LsrR, transcriptional repressor of lsr operon
tcp	Methyl-accepting chemotaxis protein I	*tcp*	Methyl-accepting chemotaxis protein I
tsr	Methyl-accepting chemotaxis protein I	*tsr*	Methyl-accepting chemotaxis protein I
SEE1_3213	Putative methyl-accepting chemotaxis protein	*SEE2_3209*	Putative methyl-accepting chemotaxis protein
SEE1_3228	Methyl-accepting chemotaxis protein I	*SEE2_3224*	Methyl-accepting chemotaxis protein I

Table S13. SEE1/SEE2 Antimicrobial Resistance Genes

SEE1		SEE2	
**Gene**	**Description**	**Gene**	**Description**
yadG	ABC-type multidrug transport system, ATPase component	*yadG*	ABC-type multidrug transport system, ATPase component
yadH	ABC-type multidrug transport system, permease component	*yadH*	ABC-type multidrug transport system, permease component
SEE1_0272	Putative drug efflux protein	*SEE2_0271*	Putative drug efflux protein
mod	Type III restriction-modification system methylation subunit	*mod*	Type III restriction-modification system methylation subunit
res	Type III restriction-modification system StyLTI enzyme res	*res*	Type III restriction-modification system StyLTI enzyme res
SEE1_0352	RND efflux system, outer membrane lipoprotein CmeC	*SEE2_0351*	RND efflux system, outer membrane lipoprotein CmeC
SEE1_0353	RND efflux system, inner membrane transporter CmeB	*SEE2_0352*	RND efflux system, inner membrane transporter CmeB
SEE1_0354	RND efflux system, membrane fusion protein CmeA	*SEE2_0353*	RND efflux system, membrane fusion protein CmeA
ampG	AmpG permease	*ampG*	AmpG permease
acrB	RND efflux system, inner membrane transporter CmeB	*acrB*	RND efflux system, inner membrane transporter CmeB
acrA	Membrane fusion protein of RND family multidrug efflux pump	*acrA*	Membrane fusion protein of RND family multidrug efflux pump
acrR	Transcription repressor of multidrug efflux pump acrAB operon	*acrR*	Transcription repressor of multidrug efflux pump acrAB operon
fsr	Fosmidomycin resistance protein	*fsr*	Fosmidomycin resistance protein
copA	Lead, cadmium, zinc and mercury transporting ATPase. Copper-translocating P-type ATPase	*copA*	Lead, cadmium, zinc and mercury transporting ATPase. Copper-translocating P-type ATPase
yfdH	Polymyxin resistance protein ArnC, glycosyl transferase	*yfdH*	Polymyxin resistance protein ArnC, glycosyl transferase
SEE1_0388	Possible efflux pump	*SEE2_0387*	Possible efflux pump
SEE1_0575	Cobalt-zinc-cadmium resistance protein CzcA; Cation efflux system protein CusA	*SEE2_0574*	Cobalt-zinc-cadmium resistance protein CzcA; Cation efflux system protein CusA
ybgL	Lactam utilization protein LamB	*ybgL*	Lactam utilization protein LamB
ybgR	Cobalt-zinc-cadmium resistance protein CzcD	*ybgR*	Cobalt-zinc-cadmium resistance protein CzcD
ybhF	ABC transporter multidrug efflux pump, fused ATP-binding domains	*ybhF*	ABC transporter multidrug efflux pump, fused ATP-binding domains
SEE1_0812	Predicted membrane fusion protein component of efflux pump,	*SEE2_0809*	Predicted membrane fusion protein component of efflux pump,

ybiH	Transcriptional regulator YbiH, TetR family	*ybiH*	Transcriptional regulator YbiH, TetR family
mdfA	Multidrug translocase MdfA	*mdfA*	Multidrug translocase MdfA
ybjY	Macrolide-specific efflux protein MacA	*ybjY*	Macrolide-specific efflux protein MacA
ybjZ	Macrolide export ATP-binding/permease protein MacB	*ybjZ*	Macrolide export ATP-binding/permease protein MacB
yobA	Copper resistance protein C precursor	*yobA*	Copper resistance protein C precursor
SEE1_1216	Copper resistance protein D	*SEE2_1211*	Copper resistance protein D
SEE1_1461	Probable transcription regulator protein of MDR efflux pump cluster	*SEE2_1456*	Probable transcription regulator protein of MDR efflux pump cluster
SEE1_1510	Ethidium bromide-methyl viologen resistance protein EmrE	*SEE2_1504*	Ethidium bromide-methyl viologen resistance protein EmrE
yedA	Permease of the drug/metabolite transporter	*yedA*	Permease of the drug/metabolite transporter
tehA	Tellurite resistance protein TehA	*tehA*	Tellurite resistance protein TehA
tehB	Tellurite resistance protein TehB	*tehB*	Tellurite resistance protein TehB
smvA	Methyl viologen resistance protein smvA	*smvA*	Methyl viologen resistance protein smvA
yddG	Permease of the drug/metabolite transporter	*yddG*	Permease of the drug/metabolite transporter
SEE1_1623	12-TMS multidrug efflux protein homolog	*SEE2_1616*	12-TMS multidrug efflux protein homolog
marC	Multiple antibiotic resistance protein MarC	*marC*	Multiple antibiotic resistance protein MarC
marR	Multiple antibiotic resistance protein MarR	*marR*	Multiple antibiotic resistance protein MarR
marA	Multiple antibiotic resistance protein MarA	*marA*	Multiple antibiotic resistance protein MarA
marB	Multiple antibiotic resistance protein MarB	*marB*	Multiple antibiotic resistance protein MarB
ydeD	Permease of the drug/metabolite transporter	*ydeD*	Permease of the drug/metabolite transporter
SEE1_1684	Acid shock protein precursor	*SEE2_1677*	Acid shock protein precursor
katE	Catalase	*katE*	Catalase
yceE	Multidrug-efflux transporter, major facilitator superfamily	*yceE*	Multidrug-efflux transporter, major facilitator superfamily
msyB	Acidic protein msyB	*msyB*	Acidic protein msyB
yegN	Multidrug transporter MdtB	*yegN*	Multidrug transporter MdtB
yegO	Multidrug transporter MdtC	*yegO*	Multidrug transporter MdtC
yegB	Multidrug transporter MdtD	*yegB*	Multidrug transporter MdtD
yohG	RND efflux system, outer membrane lipoprotein, NodT family	*yohG*	RND efflux system, outer membrane lipoprotein, NodT family

bcr	MFS family multidrug transport protein, bicyclomycin resistance protein	*bcr*	MFS family multidrug transport protein, bicyclomycin resistance protein
ais	Polymyxin resistance protein PmrG; Ais protein	*ais*	Polymyxin resistance protein PmrG; Ais protein
pmrF	Polymyxin resistance protein ArnC, glycosyl transferase	*pmrF*	Polymyxin resistance protein ArnC, glycosyl transferase
yfbG	Polymyxin resistance protein ArnA_DH, UDP-glucuronic acid decarboxylase	*yfbG*	Polymyxin resistance protein ArnA_DH, UDP-glucuronic acid decarboxylase
pmrJ	Polymyxin resistance protein PmrJ, predicted deacetylase	*pmrJ*	Polymyxin resistance protein PmrJ, predicted deacetylase
pqaB	Polymyxin resistance protein ArnT, undecaprenyl phosphate-alpha-L-Ara4N transferase;	*pqaB*	Polymyxin resistance protein ArnT, undecaprenyl phosphate-alpha-L-Ara4N transferase;
pmrL	Polymyxin resistance protein PmrL, sucrose-6 phosphate hydrolase	*pmrL*	Polymyxin resistance protein PmrL, sucrose-6 phosphate hydrolase
pmrM	Polymyxin resistance protein PmrM	*pmrM*	Polymyxin resistance protein PmrM
pmrD	Polymyxin resistance protein PmrD	*pmrD*	Polymyxin resistance protein PmrD
emrR	Transcription repressor	*emrR*	Transcription repressor
emrA	Multidrug resistance protein A	*emrA*	Multidrug resistance protein A
emrB	Inner membrane component of tripartite multidrug resistance system	*emrB*	Inner membrane component of tripartite multidrug resistance system
mdaB	Modulator of drug activity B	*mdaB*	Modulator of drug activity B
ygjT	Integral membrane protein TerC	*ygjT*	Integral membrane protein TerC
envR	Transcription repressor of multidrug efflux pump acrAB operon	*envR*	Transcription repressor of multidrug efflux pump acrAB operon
acrE	RND efflux system, membrane fusion protein CmeA	*acrE*	RND efflux system, membrane fusion protein CmeA
acrF	RND efflux system, inner membrane transporter CmeB	*acrF*	RND efflux system, inner membrane transporter CmeB
damX	DamX, an inner membrane protein involved in bile resistance	*damX*	DamX, an inner membrane protein involved in bile resistance
emrD	Multidrug resistance protein D	*emrD*	Multidrug resistance protein D
zntA	Lead, cadmium, zinc and mercury transporting ATPase Copper-translocating P-type ATPase	*zntA*	Lead, cadmium, zinc and mercury transporting ATPase Copper-translocating P-type ATPase
aadA	Streptomycin 3''-O-adenylyltransferase/Spectinomycin 9-O-adenylyltransferase	*aadA*	Streptomycin 3''-O-adenylyltransferase/Spectinomycin 9-O-adenylyltransferase
SEE1_3829	Putative beta-lactamase	*SEE2_3822*	Putative beta-lactamase

katG	Catalase/Peroxidase	*katG*	Catalase/Peroxidase
oxyR	Hydrogen peroxide-inducible genes activator	*oxyR*	Hydrogen peroxide-inducible genes activator
sugE	Quaternary ammonium compound-resistance protein SugE	*sugE*	Quaternary ammonium compound-resistance protein SugE

Table S14. SEE1/SEE2 Miscellaneous/Putative Virulence Genes

SEE1		SEE2	
**Gene**	**Description**	**Gene**	**Description**
SEE1_0088	Probable secreted protein	*SEE2_0087*	Probable secreted protein
SEE1_0089	Probable secreted protein	*SEE2_0088*	Probable secreted protein
SEE1_0336	Probable secreted protein	*SEE2_0307*	Probable secreted protein
SEE1_0350	Probable secreted protein	*SEE2_0349*	Probable secreted protein
hha	Haemolysin expression modulating protein	*hha*	Haemolysin expression modulating protein
ybaJ	FIG00948312: hypothetical protein	*ybaJ*	FIG00948312: hypothetical protein
yliH	Biofilm regulator BssR	*yliH*	Biofilm regulator BssR
SEE1_0512	Probable secreted protein	*SEE2_0511*	Probable secreted protein
SEE2_1070	Putative exported protein	*SEE2_1066*	Putative exported protein
SEE2_1072	Putative exported protein	*SEE2_1068*	Putative exported protein
SEE1_1073	Secreted protein Hcp	*SEE2_1070*	Secreted protein Hcp
rcsA	Colanic acid capsular biosynthesis activation accesory protein RcsA	*rcsA*	Colanic acid capsular biosynthesis activation accesory protein RcsA
ycgB	Stage V sporulation protein involved in spore cortex synthesis (SpoVR)	*ycgB*	Stage V sporulation protein involved in spore cortex synthesis (SpoVR)
opgD	Glucans biosynthesis protein D precursor	*opgD*	Glucans biosynthesis protein D precursor
ybgS	Probable secreted protein	*ybgS*	Probable secreted protein
mdoH	Glucans biosynthesis glucosyltransferase H	*mdoH*	Glucans biosynthesis glucosyltransferase H
mdoG	Glucans biosynthesis protein G precursor	*mdoG*	Glucans biosynthesis protein G precursor
mdoC	Glucans biosynthesis protein C	*mdoC*	Glucans biosynthesis protein C
galF	UTP--glucose-1-phosphate uridylyltransferase	*galF*	UTP--glucose-1-phosphate uridylyltransferase
wcaM	Colanic acid biosynthesis protein wcaM	*wcaM*	Colanic acid biosynthesis protein wcaM
wcaL	Colanic acid biosynthesis glycosyl transferase WcaL	*wcaL*	Colanic acid biosynthesis glycosyl transferase WcaL
wcaK	Colanic acid biosysnthesis protein WcaK	*wcaK*	Colanic acid biosysnthesis protein WcaK
wzxZ	Lipopolysaccharide biosynthesis protein WzxC	*wzxZ*	Lipopolysaccharide biosynthesis protein WzxC
wcaJ	Colanic acid biosynthsis UDP-glucose lipid carrier transferase WcaJ	*wcaJ*	Colanic acid biosynthsis UDP-glucose lipid carrier transferase WcaJ

cpsG	Phosphomannomutase	*cpsG*	Phosphomannomutase
manC	Mannose-1-phosphate guanylyltransferase/Mannose-6-phosphate isomerase	*manC*	Mannose-1-phosphate guanylyltransferase/Mannose-6-phosphate isomerase
wcaI	Colanic acid biosysnthesis glycosyl transferase WcaI	*wcaI*	Colanic acid biosysnthesis glycosyl transferase WcaI
wcaH	GDP-mannose mannosyl hydrolase	*wcaH*	GDP-mannose mannosyl hydrolase
wcaG	GDP-L-fucose synthetase/Colanic acid biosynthesis protein wcaG	*wcaG*	GDP-L-fucose synthetase/Colanic acid biosynthesis protein wcaG
gmd	GDP-mannose 4,6-dehydratase	*gmd*	GDP-mannose 4,6-dehydratase
wcaF	Colanic acid biosynthesis acetyltransferase WcaF	*wcaF*	Colanic acid biosynthesis acetyltransferase WcaF
wcaE	Colanic acid biosynthesis glycosyl transferase WcaE	*wcaE*	Colanic acid biosynthesis glycosyl transferase WcaE
wcaD	Colanic acid polymerase WcaD	*wcaD*	Colanic acid polymerase WcaD
wcaC	Colanic acid biosynthesis glycosyl transferase WcaC	*wcaC*	Colanic acid biosynthesis glycosyl transferase WcaC
wcaB	Colanic acid biosynthesis acetyltransferase WcaB	*wcaB*	Colanic acid biosynthesis acetyltransferase WcaB
wcaA	Putative N-acetylgalactosaminyl-diphosphoundecaprenol glucuronosyltransferase	*wcaA*	Putative N-acetylgalactosaminyl-diphosphoundecaprenol glucuronosyltransferase
wzc	Tyrosine-protein kinase Wzc	*wzc*	Tyrosine-protein kinase Wzc
wzb	Low molecular weight protein-tyrosine-phosphatase Wzb	*wzb*	Low molecular weight protein-tyrosine-phosphatase Wzb
wza	Polysaccharide export lipoprotein Wza	*wza*	Polysaccharide export lipoprotein Wza
yegH	Putative capsular polysaccharide transport protein YegH	*yegH*	Putative capsular polysaccharide transport protein YegH
SEE1_2653	Alpha-2-macroglobulin	*SEE2_2651*	Alpha-2-macroglobulin
SEE1_4402	Entericidin B precursor	*SEE2_4396*	Entericidin B precursor
cvpA	Colicin V production protein	*cvpA*	Colicin V production protein
SEE1_3870	Probable secreted protein STY4010	*SEE2_3863*	Probable secreted protein STY4010
yiaF	Probable exported protein YPO4070	*yiaF*	Probable exported protein YPO4070
SEE1_2892	Putative exported protein	*SEE2_2889*	Putative exported protein
SEE1_2893	Putative exported protein	*SEE2_2890*	Putative exported protein

yfbK	Von Willebrand factor type A domain protein	*yfbK*	Von Willebrand factor type A domain protein
yebW	Putative secreted protein YebW	*yebW*	Putative secreted protein YebW
ynfB	Putative secreted protein	*ynfB*	Putative secreted protein
SEE1_1375	Putative secreted protein	*SEE2_1370*	Putative secreted protein
SEE1_1545	Putative exported protein	*SEE2_1539*	Putative exported protein
eutR	Ethanolamine operon regulatory protein	*eutR*	Ethanolamine operon regulatory protein
eutK	Ethanolamine utilization polyhedral-body-like protein EutK	*eutK*	Ethanolamine utilization polyhedral-body-like protein EutK
eutL	Ethanolamine utilization polyhedral-body-like protein EutL	*eutL*	Ethanolamine utilization polyhedral-body-like protein EutL
eutC	Ethanolamine ammonia-lyase light chain	*eutC*	Ethanolamine ammonia-lyase light chain
eutB	Ethanolamine ammonia-lyase heavy chain	*eutB*	Ethanolamine ammonia-lyase heavy chain
eutA	Ethanolamine utilization protein EutA	*eutA*	Ethanolamine utilization protein EutA
eutH	Ethanolamine permease	*eutH*	Ethanolamine permease
eutG	Ethanolamine utilization protein EutG	*eutG*	Ethanolamine utilization protein EutG
eutJ	Ethanolamine utilization protein EutJ	*eutJ*	Ethanolamine utilization protein EutJ
eutE	Acetaldehyde dehydrogenase, ethanolamine utilization cluster	*eutE*	Acetaldehyde dehydrogenase, ethanolamine utilization cluster
eutN	Ethanolamine utilization polyhedral-body-like protein EutN	*eutN*	Ethanolamine utilization polyhedral-body-like protein EutN
eutM	Ethanolamine utilization polyhedral-body-like protein EutM	*eutM*	Ethanolamine utilization polyhedral-body-like protein EutM
eutD	Phosphate acetyltransferase, ethanolamine utilization-specific	*eutD*	Phosphate acetyltransferase, ethanolamine utilization-specific
eutT	ATP:Cob(I)alamin adenosyltransferase, ethanolamine utilization	*eutT*	ATP:Cob(I)alamin adenosyltransferase, ethanolamine utilization
eutQ	Ethanolamine utilization protein EutQ	*eutQ*	Ethanolamine utilization protein EutQ
eutP	Ethanolamine utilization protein EutP	*eutP*	Ethanolamine utilization protein EutP

eutS	Ethanolamine utilization polyhedral-body-like protein EutS	*eutS*	Ethanolamine utilization polyhedral-body-like protein EutS
SEE1_0285	Rhs-Family Protein	*SEE2_0284*	Rhs-Family Protein
SEE1_0289	rhs core protein with extension	*SEE2_0288*	rhs core protein with extension

Table S15. Core Conserved Adherence and OMP COGs in *Salmonella enterica*

COG#	Protein ID	Description
COG1178:	229037912	autoagglutination protein
COG1193:	62178590	fimbrial subunit
COG1194:	62178591	fimbrial chaperone
COG1195:	62178592	fimbrial subunit
COG1196:	62178593	fimbrial subunit
COG1197:	62178595	fimbrial chaperone
COG1227:	62178645	outer membrane lipoprotein
COG1273:	62178711	type IV pilin biogenesis protein
COG1275:	62178713	major pilin subunit
COG1308:	62178776	vitamin B12-transporter protein BtuF
COG1339:	62178813	outer membrane lipoprotein
COG1352:	62178877	adhesin
COG1368:	62178952	fimbriae chaperone
COG1369:	62178953	fimbriae major subunit
COG1374:	62178962	outer membrane lipoprotein
COG1390:	62178988	outer membrane lipoprotein
COG1509:	62179155	outer membrane usher protein
COG1510:	62179156	minor fimbrial subunit
COG1511:	62179157	fimbrial protein
COG1514:	62179160	fimbrial protein
COG1521:	62179178	N-acetyl phenylalanine beta-naphthyl ester-cleaving esterase
COG1587:	62179278	outer membrane protein
COG1672:	62179399	outer membrane protein X
COG1766:	62179526	outer membrane protein 1a (IA;b;f), porin
COG1777:	62179587	outer membrane protein
COG1781:	62179592	outer membrane protein OmpA
COG1798:	62179613	outer protein
COG1821:	62179651	outer membrane protein
COG1871:	62179726	outer membrane lipoprotein
COG1872:	62179727	outer membrane lipoprotein
COG1878:	62179734	outer membrane protein
COG1906:	62179836	outer membrane lipoprotein
COG1959:	62179919	outer membrane protein
COG2034:	62180033	outer membrane lipoprotein
COG2053:	62180060	outer membrane protein N, non-specific porin
COG2082:	62180115	outer membrane protein

COG2093:	62180174	outer membrane lipoprotein
COG2110:	62180210	outer membrane lipoprotein
COG2220:	62180383	outer membrane protein
COG2247:	62180433	PhoPQ-activated integral membrane protein
COG2294:	62180510	outer membrane lipoprotein
COG2419:	62180737	fimbrial-like protein
COG2464:	62180800	outer membrane lipoprotein
COG2590:	62180966	outer membrane protease
COG2652:	62181083	outer membrane protein
COG2925:	62181563	outer membrane protein
COG3003:	62181690	outer membrane protein
COG3134:	62181869	outer membrane protein
COG3156:	62181900	outer membrane lipoprotein
COG3484:	62182415	outer membrane lipoprotein
COG3572:	62182558	outer membrane lipoprotein
COG3627:	62182672	outer membrane lipoprotein
COG3628:	62182674	outer membrane lipoprotein
COG3649:	62182702	outer membrane lipoprotein
COG3706:	62182788	outer membrane lipoprotein Blc
COG3834:	62183007	fimbrial subunit
COG3835:	62183010	fimbrial chaperone protein

Table S16. Core Conserved SPI COGs in *Salmonella enterica*

COG#	Protein ID	Description
COG1795:	62179609	pathogenicity island encoded protein: SPI3
COG1796:	62179610	pathogenicity island encoded protein: SPI3
COG1988:	62179981	MerR family transcriptional regulator
COG1989:	62179982	secretion system transcriptonal activator
COG1990:	62179983	secretion system regulator:sensor component
COG1991:	62179985	secretion system apparatus protein SsaC
COG1992:	62179986	secretion system apparatus protein SsaD
COG1993:	62179987	secretion system effector protein SsaE
COG1994:	62179989	secretion system effector protein SseB
COG1995:	62179990	secretion system chaperone protein SscA
COG1996:	62179991	secretion system effector protein SseC
COG1997:	62179992	secretion system effector protein SseD
COG1998:	62179993	secretion system effector SseE
COG1999:	62179994	secretion system chaperone protein SscB
COG2000:	62179995	secretion system effector protein SseF
COG2001:	62179996	secretion system effector protein SseG
COG2002:	62179997	secretion system apparatus protein SsaG
COG2003:	62179998	secretion system apparatus protein SsaH
COG2004:	62179999	secretion system apparatus protein SsaI
COG2005:	62180000	secretion system apparatus protein SsaJ
COG2006:	62180001	hypothetical protein SC1431
COG2007:	62180002	secretion system apparatus protein SsaK
COG2008:	62180003	secretion system apparatus protein SsaL
COG2009:	62180004	secretion system apparatus protein SsaM
COG2010:	62180005	secretion system apparatus protein SsaV
COG2011:	62180006	type III secretion system ATPase
COG2012:	62180007	secretion system apparatus protein SsaO
COG2013:	62180008	secretion system apparatus protein SsaP
COG2014:	62180009	type III secretion system protein
COG2015:	62180010	type III secretion system protein
COG2016:	62180011	secretion system apparatus protein SsaS
COG2017:	62180012	secretion system apparatus protein SsaT
COG2018:	62180013	secretion system apparatus protein SsaU
COG2810:	62181373	cell invasion protein
COG2811:	62181374	cell invasion protein
COG2812:	62181375	cell invasion protein
COG2813:	62181376	cell invasion protein

COG2814:	62181377	regulatory protein
COG2815:	62181378	invasion protein regulator
COG2816:	62181379	cell invasion protein
COG2817:	62181381	virulence associated chaperone
COG2818:	62181383	acyl carrier protein
COG2819:	62181384	cell invasion protein
COG2820:	62181385	cell invasion protein
COG2821:	62181386	cell invasion protein
COG2822:	62181387	cell invasion protein
COG2823:	62181388	surface presentation of antigens; secretory proteins
COG2824:	62181389	surface presentation of antigens protein SpaS
COG2825:	62181390	surface presentation of antigens; secretory proteins
COG2826:	62181391	surface presentation of antigens; secretory proteins
COG2827:	62181392	surface presentation of antigens protein SpaP
COG2828:	62181393	surface presentation of antigens protein SpaO
COG2829:	62181394	surface presentation of antigens; secretory proteins
COG2830:	62181395	surface presentation of antigens; secretory proteins
COG2831:	62181396	ATP synthase SpaL
COG2832:	62181397	surface presentation of antigens; secretory proteins
COG2833:	62181398	invasion protein
COG2834:	62181399	invasion protein
COG2835:	62181400	invasion protein; outer membrane
COG2836:	62181401	invasion protein
COG2837:	62181402	invasion protein

Table S17. Core Conserved LPS Biosynthesis COGs in *Salmonella enterica*

COG#	Protein ID	Description
COG1258:	62178690	UDP-N-acetylmuramoylalanyl-D-glutamate--2,6-diaminopimelate ligase
COG1259:	62178691	UDP-N-acetylmuramoyl-tripeptide--D-alanyl-D-alanine ligase
COG1260:	62178692	phospho-N-acetylmuramoyl-pentapeptide-transferase
COG1261:	62178693	UDP-N-acetylmuramoyl-L-alanyl-D-glutamate synthetase
COG1262:	62178696	UDP-N-acetylmuramate--L-alanine ligase
COG1324:	62178796	UDP-3-O-
COG1325:	62178797	(3R)-hydroxymyristoyl-ACP dehydratase
COG1326:	62178798	UDP-N-acetylglucosamine acyltransferase
COG1327:	62178799	lipid-A-disaccharide synthase
COG1504:	62179144	UDP-2,3-diacylglucosamine hydrolase
COG2265:	62180467	lipid A biosynthesis (KDO)2-(lauroyl)-lipid IVA acyltransferase
COG2595:	62180972	lipid A biosynthesis palmitoleoyl acyltransferase
COG3468:	62182394	lipopolysaccharide biosynthesis protein WzzE
COG3469:	62182395	UDP-N-acetyl glucosamine -2-epimerase
COG3470:	62182397	dTDP-glucose 4,6-dehydratase
COG3471:	62182399	TDP-fucosamine acetyltransferase
COG3472:	62182400	TDP-4-oxo-6-deoxy-D-glucose transaminase
COG3473:	62182401	O-antigen translocase in LPS biosyntesis

Table S18. Core Conserved Transporter COGs in *Salmonella enterica*

COG#	Protein ID	Description
COG1159:	162139617	iron-hydroxamate transporter ATP-binding subunit
COG1308:	62178776	vitamin B12-transporter protein BtuF
COG1379:	62178976	transporter
COG1389:	62178987	transporter
COG1528:	62179187	enterobactin/ferric enterobactin esterase
COG1530:	62179191	iron-enterobactin transporter ATP-binding protein
COG1531:	62179193	iron-enterobactin transporter membrane protein
COG1532:	62179194	enterobactin exporter EntS
COG1533:	62179195	enterobactin transporter periplasmic binding protein
COG2293:	62180508	ferritin
COG2801:	62181364	iron transporter: fur regulated
COG2802:	62181365	iron transporter: fur regulated
COG2803:	62181366	iron transporter: fur regulated
COG2804:	62181367	iron transporter: fur regulated
COG3237:	62182007	ferrous iron transport protein A
COG3238:	62182008	ferrous iron transport protein B
COG3477:	62182405	transporter
COG3654:	62182708	ABC transporter outer membrane protein
COG3656:	62182711	bacteriocin/lantibiotic ABC transporter

Table S19. Core Conserved Signaling and Motility COGs in *Salmonella enterica*

COG#	Protein ID	Description
COG1047:	162139591	methyl-accepting chemotaxis protein
COG1048:	62178617	transcription regulator, histidine kinase for citrate
COG1128:	162139574	chemotaxis regulator CheZ
COG1464:	62179085	hemolysin expression-modulating protein
COG1845:	62179688	flagellar biosynthesis chaperone
COG1846:	62179689	anti-sigma-28 factor FlgM
COG1847:	62179690	flagellar basal body P-ring biosynthesis protein FlgA
COG1848:	62179691	flagellar basal-body rod protein FlgB
COG1849:	62179692	flagellar basal body rod protein FlgC
COG1850:	62179693	flagellar basal body rod modification protein
COG1851:	62179694	flagellar hook protein FlgE
COG1852:	62179695	flagellar basal body rod protein FlgF
COG1853:	62179696	flagellar basal body rod protein FlgG
COG1854:	62179697	flagellar basal body L-ring protein
COG1855:	62179699	flagellar rod assembly protein/muramidase FlgJ
COG1856:	62179701	flagellar hook-associated protein FlgL
COG1894:	62179751	sensor protein PhoQ
COG1895:	62179752	DNA-binding transcriptional regulator PhoP
COG2051:	62180058	sensor protein RstB
COG2286:	62180498	chemotaxis protein CheA
COG2308:	62180529	flagella biosynthesis protein FliZ
COG2309:	62180530	flagellar biosynthesis sigma factor
COG2310:	62180534	flagellar capping protein
COG2311:	62180535	flagellar protein FliS
COG2312:	62180536	flagellar biosynthesis protein FliT
COG2318:	62180542	flagellar hook-basal body protein FliE
COG2319:	62180544	flagellar MS-ring protein
COG2320:	62180545	flagellar motor switch protein G
COG2321:	62180546	flagellar assembly protein H
COG2322:	62180547	flagellum-specific ATP synthase
COG2323:	62180548	flagellar biosynthesis chaperone
COG2324:	62180549	flagellar hook-length control protein
COG2325:	62180551	flagellar motor switch protein FliM
COG2326:	62180553	flagellar biosynthesis protein FliO
COG2327:	62180554	flagellar biosynthesis protein FliP
COG2328:	62180555	flagellar biosynthesis protein FliQ
COG2329:	62180556	flagellar biosynthesis protein FliR
COG2408:	62180702	signal transduction histidine-protein kinase BaeS

COG2409:	62180703	DNA-binding transcriptional regulator BaeR
COG2424:	62180744	two-component response-regulatory protein YehT
COG2425:	62180745	sensor/kinase in regulatory system
COG2521:	62180884	chemotaxis signal transduction protein
COG2687:	62181127	transcriptional regulator of two-component regulator protein
COG2689:	62181129	sensory kinase in regulatory system
COG2736:	62181258	SsrA-binding protein
COG2808:	62181371	flagellar biosynthesis/type III secretory pathway protein
COG2866:	62181468	hybrid sensory histidine kinase BarA
COG3004:	62181691	DNA-binding transcriptional regulator QseB
COG3005:	62181692	sensor protein QseC
COG3035:	62181731	transcriptional regulator
COG3036:	62181732	methyl-accepting chemotaxis protein
COG3037:	62181733	aerotaxis sensor receptor
COG3234:	62182003	osmolarity sensor protein
COG3235:	62182004	osmolarity response regulator
COG3546:	62182519	two-component sensor protein
COG3547:	62182520	DNA-binding transcriptional regulator CpxR
COG3559:	62182538	autoinducer-2 (AI-2) modifying protein LsrG
COG3613:	62182624	sensor protein ZraS
COG3614:	62182625	transcriptional regulatory protein ZraR
COG3653:	62182707	methyl-accepting chemotaxis protein
COG3654:	62182708	ABC transporter outer membrane protein
COG3656:	62182711	bacteriocin/lantibiotic ABC transporter
COG3680:	62182740	sensor protein BasS/PmrB
COG3681:	62182741	DNA-binding transcriptional regulator BasR
COG3731:	62182823	biofilm stress and motility protein A
COG3831:	62183003	DNA-binding response regulator CreB
COG3832:	62183004	sensory histidine kinase CreC
COG3837:	62183013	two-component response regulator

Table S20. Core Conserved Miscellaneous/Survival COGs in *Salmonella enterica*

COG#	Protein ID	Description
COG1647:	62179362	biotin synthetase
COG1724:	62179465	virK
COG1844:	62179687	virulence factor
COG1888:	62179744	secreted effector protein
COG1903:	62179822	macrophage survival gene; reduced mouse virulence
COG1923:	62179856	hemolysin
COG1965:	62179928	integration host factor subunit alpha
COG1985:	62179978	tetrathionate reductase complex: response regulator
COG2025:	62180020	superoxide dismutase
COG2031:	62180029	superoxide dismutase
COG2330:	62180557	capsular/exo- polysaccharide synthesis transcriptional regulator
COG2390:	62180673	colanic acid exporter
COG2391:	62180677	glycosyl transferase family protein
COG2392:	62180678	glycosyl transferase in colanic acid biosynthesis
COG2393:	62180680	GDP-D-mannose dehydratase
COG2394:	62180681	colanic acid biosynthesis acetyltransferase WcaF
COG2395:	62180682	glycosyl transferase family protein
COG2396:	62180684	glycosyl transferase family protein
COG2397:	62180685	colanic acid biosynthesis acetyltransferase WcaB
COG2623:	62181027	transport protein in ethanolamine utilization
COG2627:	62181034	ethanolamine utilization protein
COG2628:	62181035	ethanolamine utilization protein
COG2738:	62181265	HlyD family secretion protein
COG2742:	62181282	virulence protein VirK
COG3544:	62182516	superoxide dismutase
COG3731:	62182823	biofilm stress and motility protein A
